# Loss factor and moisture diffusivity property estimation of lentil crop during microwave processing

**DOI:** 10.1016/j.crfs.2021.12.008

**Published:** 2021-12-25

**Authors:** Mohamad Mehdi Heydari, Tahereh Najib, Oon-Doo Baik, Kaiyang Tu, Venkatesh Meda

**Affiliations:** a Department of Chemical and Biological Engineering, University of Saskatchewan, 57 Campus Drive, Saskatoon, SK, S7N 5A9, Canada; b Canadian Light Source Inc., 44 Innovation Boulevard, Saskatoon, SK S7N 2V3, Canada

**Keywords:** Loss factor, Effective moisture diffusivity, Heat and mass transfer, Thermal properties, FT-MIR spectroscopy

## Abstract

Characterization of loss factor and moisture diffusivity are required to understand materials' precise behavior during microwave processing. However, providing the processing facilities to measure these properties in a real or simulated situation directly can be complicated or unachievable. Hence, this study proposes an alternative procedure for modeling these properties according to their affecting factors including temperature, and moisture content. The basis of this method is to use an algorithm that combines the optimization approach and the numerical solution of the heat and mass transfer governing equations, including boundary conditions. For this aim, the coefficients of estimated models for loss factor and moisture diffusivity were obtained by minimizing the sum square error of the experimentally measured mean surface temperature and moisture content and the predicted values by solving the system of partial differential equations. The suggested models illustrated that during the microwave process, the moisture diffusivity grows arithmetically, and the loss factor generally raises, but transition points were observed in the trend for the samples tempered up to the 50% moisture content. These points have been attributed to the starch gelatinization and confirm how the bio-chemical reaction would have a noticeable effect on this property, determining the microwave energy absorbance. The results of differential scanning calorimetry thermograms and the Fourier transform mid-infrared spectra of flours obtained from microwave processed lentil seeds also confirmed the greatest intensity of starch structure alteration happened for the samples tempered to 50% moisture content by showing the highest shifts in the endothermic peak and lowest degree of order.

## Highlights

• Combination multiobjective optimization and heat and mass transfer simulation.
• Estimating models for loss factor and moisture diffusivity variations.
• Predicting the effect of starch gelatinization on loss factor property by the model.
• DSC and FT-MIR confirmed the various intensities in starch structure alteration.

## Graphical abstract

**Figure:**
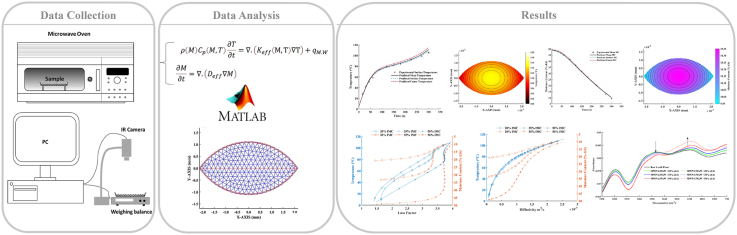

## 1 Introduction

Microwave heating has attracted great interest in the food processing industry as an alternative to the conventional heating method. The rapid and volumetric heating capabilities of microwaves are extensively recognized and often advanced as explanations for their superior energy performance over conventional heaters (Atuonwu and Tassou, 2019). Therefore, the reduction in food products' thermal processing during microwave treatment can preserve the product quality (Pham et al., 2020; Zhang et al., 2014).

Moisture content, temperature, and chemical compositions of a food item, as well as the frequency of applied microwave, are the factors affecting the values of dielectric properties determining the amount of absorbed electromagnetic energy as heat (Taheri et al., 2019). Hence, during the microwave treatment of a product, the mass and heat transfer are happening simultaneously, and changes in the food item's dielectric properties are not beyond the expectation. These phenomena clearly illustrate the need to have a function that explains the variation of food dielectric properties according to the temperature and moisture content of material under irradiation. Several studies try to establish the regression models for variation in dielectric properties of fruits, vegetables, and grains matching the affecting factors (Calay et al., 1994; Guo et al., 2010; Jiao et al., 2011; Sipahioglu and Barringer, 2003). However, there is a limitation in the range of their measurements for affecting factors, which is reasonable because of the difficulty and complexity to create some combination of these factors.

Moreover, it needs to be considered that a quick increase in temperature during microwave heating brings about some chemical reactions such as Maillard reaction, starch gelatinization, and protein denaturation, which lead to altering the structure of chemical components of the treated commodity (Ahmed et al., 2021; Hoover et al., 2010; Ma et al., 2011). By affecting the mobility and role of small polar water molecules or ionic conduction, this shift in the structure of chemical composition will also have a direct impact on the dielectric properties and the ability of microwave absorption. This phenomenon clearly represents the requirement of a real-time monitoring system of these properties to have efficient control of the microwave process. For instance, the necessity of such a system for starchy food has been recommended by the researchers (Tao et al., 2020). However, finding the methods or designing a facility for such a purpose can be a challenging task in this field.

Another parameter affected by the rapid changes in temperature and moisture content during microwave processing is the effective moisture diffusivity. This property explains all probable mechanisms of mass transfer inside the foods during dehydration or adsorption processes, including liquid diffusion, vapor diffusion, capillary flow, or hydrodynamic flow (Dadalı et al., 2007). Having an equation that describes variation in the effective moisture diffusivity of food during the microwave process is also a necessity to accurately model and estimate the mass transfer during this process, and similar to the dielectric property's measurement, it needs a great deal of energy and effort to gather data and obtain a model for effective diffusion coefficient which can cover a wide range of temperatures and moisture contents.

On the other hand, it can be stated that microwave treatment is a special treatment because it can show us the behavior of a food product in a wide range of changing temperatures and moisture contents in a short time. Therefore, instead of an excessive investment of energy and time to gather data and obtain the equations covering variation of loss factor (dielectric property) and moisture diffusivity with temperature and moisture content to use in the analytical model of microwave treatment, these properties can be estimated by analyzing the behavior of food during microwave thermal process, so-called inverse simulation. In fact, the mathematical models of the process can be solved by initial guess of unknown parameters for the models of loss factor and moisture diffusivity, and then the appropriate parameter values can be obtained by optimizing the output of the analytical model of the system in a way to gain the minimal error in the prediction of convenient measurable factors during the process, including moisture content and item's surface temperature. This is the primary purpose of the current study, and the authors would try to explain how this aim can be achievable. Considering the importance of plant-based protein these days and the thermal treatment practicality in modifying and improving legume nutritional and functional properties, the case study in the current research is lentil seed. Thermal analysis and Fourier transform mid-infrared (FT-MIR) spectroscopy of lentil samples also have been done to understand how altering the starch structure, as the main chemical component of lentil seeds, during the microwave process affect the loss factor and to show how the model can estimate the occurrence of this phenomena.

## 2 Materials and methods

### 2.1 Lentil seed and the preparation process

The red lentil (*Lens culinaris*) used for the thermal treatment experiments was provided by Viterra Inc. (Regina, SK, Canada). The red lentil seeds were from the CDC maxim variety harvested in the 2018 farming season. The received seeds contained dirt and foreign materials removed with Forsberg Vacuum Gravity Separator (Forsbergs Inc., Thief River Falls, MN, USA) before use. The initial moisture content of lentil seeds was measured using the air oven loss-on-drying method (AOAC method 925.10), found to be 10% dry basis (d.b). Before thermal processing, seeds were tempered to three higher moisture contents, 20 ± 1, 35 ± 1, and 50 ± 1% (d.b), by adding the precalculated amount of deionized water according to the method described by Heydari et al. (2020). In brief, 100 g of lentil seeds were transferred into airtight glass jars, followed by adding the required precalculated amount of deionized water to achieve different levels of tempering values. The airtight jars were kept in the laboratory at room temperature (23 °C) for 24 h and then transferred and kept in the controlled environment room (4 °C) for 72 h before the microwave process. The jars were hand-shaken at intervals during this time to achieve uniform moisture distribution inside the seeds.

### 2.2 Thermal process procedure

Advantium™ oven (GE Company, Louisville, USA) with a cavity size of 21 cm in height, 48 cm in length, and 34 cm in width was used for the microwave heating treatment. The output power of this device can be adjusted, and two levels of 0.35 and 0.7 kW nominal output power have been set to perform drying of tempered lentil seeds until they return to their initial moisture content. During this process, the loss in weight of samples was recorded with a digital scale with an accuracy of ±0.01 g (Symmetry, PR4200, Cole-Parmer Instrument Co. Vernon Hills, IL, USA) at varied time intervals from 15 seconds to 1 min, and drying experiments were performed in triplicates. In the present studies, the drying experiments were carried out by using 50 ± 5 g of lentil seeds arranged in a perforated Teflon plate (15 cm diameter) allocated on 2 cm stands inside the microwave cavity to allow airflow at the bottom surface of the seed (schematic diagram of the microwave oven and the arrangement of sample position inside that have been shown in graphical abstract).

### 2.3 Magnetron power output measurement and electric field strength

The available power from the magnetron can be determined by using the calorimetry-based calibration method (IEC-60705, 1999), which includes warming up 1000 ± 5 g of water, loaded in a cylindrical standard glass container (external diameter 190 mm, height 90 mm), to raise its temperature from 10 ± 1 °C to 20 ± 2 °C. The calculated magnetron available power is 0.616 kW, which is 88% of the nominal rated power of the microwave, 0.7 kW. By adjusting the device outpower to 0.35 kW, half of the magnetron available power was obtained.

The magnetron output power varies by the impedance of the heating load put inside the cavity of the microwave. Knowing the exact magnetron power output is needed to have a correct estimation of electric field strength in the microwave cavity (Pitchai et al., 2012). By considering the reflection coefficient, the proper estimation of electric field strength can be obtained by Equation (1) (Pitchai et al., 2012; Soltysiak et al., 2008).

(1) $$E=\sqrt{\frac{2P_{av}}{1-|S_{11}{|}^{2}}}$$

where *Pav* is available magnetron power, E is electric field strength (V.cm ^−1^), and $S_{11}$ is the reflection coefficient. The reflection coefficient values of 0.694 and 0.421 were considered for the 0.35 and 0.7 kW microwave nominal powers, respectively (Soltysiak et al., 2008).

### 2.4 Measurements of sample surface temperature

The average surface temperature of samples was monitored and measured using a thermal infrared camera (FLIR, model: Ax5) with thermal sensitivity of 0.1 °C, which captured heat images of the sample and displayed them through FLIR GEV DEMO 1.10 software. In the camera setting section, some parameters, including sample emissivity, the distance between sample and camera, temperature, and relative humidity of the air, can be specified, which leads to more accurate measurement. The air temperature, relative humidity, and the distance between the sample and camera were 23 °C, 50%, and 30 cm, respectively. The emissivity of lentil seed is also determined based on the procedure explained by Taheri et al. (2019) and found to be in a similar average amount of 0.9. During the microwave treatment process, the surface temperature of samples was evaluated while measuring the weight loss by taking the sample out of the microwave. The whole process of measurements was done in less than 20 s (schematic diagram of measuring weight and surface temperature process of samples has been shown in graphical abstract).

### 2.5 Assumptions of the model

The following assumptions have been made to make the modeling process simple and to take into consideration the simulation accuracy and computation efficiency:

1) The lentil seed is assumed to be a homogenous and isotropic material, and the distribution of temperature and moisture content is initially uniform.
2) The simulation was developed for a kernel seed, and the average variation of moisture content and surface temperature of the drying layer was considered for its modeling.
3) Considering the lens shape of the lentil, the two-dimensional axisymmetric model, having two circular segments, was used for modeling with the average diameter and thickness of 4.4 and 2.2 mm, respectively. By rotating 180° of this shape about the vertical axis, the whole lentil shape can be derived.
4) Contribution of diffusion on moisture variation is far smaller compared to the rapid evaporation under intense microwave heating.
5) Due to the lens shape of the seed and its small contact area to the container, the probable conduction heat transfer happening at the part of the bottom surface where the seed is in contact with the container was ignored in simulation, and the same boundary conditions have been considered for the top and bottom surfaces of the seed.
6) Because the microwave penetration depth in lentils, having an average of 2 cm at 2450 MHz frequency (Taheri et al., 2018), is significantly higher than the seed thickness or drying layer, the uniform distribution of electric field was assumed inside the seed.
7) The shrinkage of lentil seed during the thermal treatment was ignored in modeling.

### 2.6 Governing equations for heat and mass transfer

The energy conservation was expressed based on Fourier's law of heat transfer, as characterized by Equation (2) (Jiang et al., 2017):

(2) $$\rho C_{p}\frac{\partial T}{\partial t}=\nabla .\left(k_{eff}\nabla \text{T}\right)+q_{M.W}-H_{evap}M_{v}$$

where *ρ* is the sample density (kg.m^−3^) and is a function of moisture content calculated from Equation (3), *C*_*p*_ is lentil specific heat (J.kg^−1^.K^−1^), which is a function of moisture content and temperature determined by Equation (4) (Tang et al., 1991), *T* is the temperature of sample (K), *t* time (s), $k_{eff}$ is the sample effective thermal conductivity (W.m^−1^.K^−1^) and evaluated by Equation (5) (Alagusundaram et al., 1991), $H_{evap}$ is the latent heat of evaporation (J.kg^−1^), determined by Equation (6) (Taheri et al., 2019), $q_{M.W}$ is the dissipated microwave power per unit volume of sample (W.m^−3^) and can be obtained by Equation (7) (Farag et al., 2012; Taheri et al., 2020), and $M_{v}$ is the volumetric evaporation rate (kg.m^−3^.s^−1^).

(3) $$\rho \left(M\right)=\left(0.193M^{2}-0.311M+1.4729\right)*10^{3}$$

(4) $$C_{p}\left(M,T\right)=\left(0.5773+0.00709\text{T}+6.22\frac{M}{1+M}-9.14{\left(\frac{M}{1+M}\right)}^{2}\right)*10^{3}$$

(5) $$k_{eff}=0.193+10^{-4}\left(T-273.15\right)+0.152\left(\frac{M}{1+M}\right)$$

(6) $$H_{evap}=2503000-2386\left(T-273.15\right)$$

(7) $$q_{M.W}=2\pi f\varepsilon_{0}\varepsilon^{"}E^{2}$$

where M is the moisture content of the sample (d.b), f is electromagnetic field frequency (2.45E9 Hz), $\varepsilon_{0}$ is the permittivity of free space (8.854E-12 F.m^−1^), $\varepsilon^{"}$ is the loss factor of the material, and E is electric field strength (V.m^−1^).

Based on the assumption (4) that the evaporation term is significantly greater than the diffusion term during the intensive thin layer microwave drying process, it can be concluded that the volumetric evaporation rate $M_{v}$ depended on the variation of moisture content (Equation (8)) (Jiang et al., 2017).

(8) $$M_{v}=-\rho \frac{\partial M}{\partial t}$$

The governing equation for moisture transport can be derived according to Fick's second law (Equation (9)) (Farag et al., 2012):

(9) $$\frac{\partial M}{\partial t}=\nabla .\left(D_{eff}\nabla M\right)$$

where $D_{eff}$ is effective moisture diffusivity (m^2^.s^−1^).

### 2.7 Equations for estimating loss factor and effective moisture diffusivity

Loss factor and effective moisture diffusivity are the remaining parameters needed to solve the system of partial differential Equations (2), (9)). This section characterizes the equations stating the relation between these parameters and affecting factors with unknown coefficients. Then in the following section, the algorithm to find these coefficients will be explained.

For the loss factor, the order of the estimated equation has been adopted according to the predictive relation proposed by Sipahioglu and Barringer (2003). In their suggested relationship, the loss factor is dependent on moisture content, temperature, and the amount of ash wet basis content. However, in this study, by accepting the fact that ash content dry basis will not significantly be altered during the thermal process, with the conversion of this value to the wet basis, Equation (10) has been used in this study.

(10) $$\varepsilon^{"}={\text{g}}_{0}+{\text{g}}_{1}{\text{T}}^{\text{′}}+{\text{g}}_{2}{{\text{T}}^{\text{′}}}^{2}+100{\text{g}}_{3}\text{M}+100{\text{g}}_{4}{\text{MT}}^{\text{′}}+{\text{g}}_{5}\frac{\text{Ash}}{\left(1+\text{M}\right)}+{\text{g}}_{6}{\left(\frac{\text{Ash}}{\left(1+\text{M}\right)}\right)}^{2}+{\text{g}}_{7}\left(\frac{\text{Ash}}{\left(1+\text{M}\right)}\right){\text{T}}^{\text{′}}$$

where, $g_{i}:\;i=\left\{0,\;2,\ldots ,\;7\right\}$ are the unknown coefficients, ${\text{T}}^{\text{′}}$ is Celsius temperature of the sample, and Ash is the percentage dry basis of ash content.

Regarding effective moisture diffusivity, the sort of anticipated equation has been developed, agreeing with the study of Husain et al. (1973). This Equation (11) consists of two parts, in which the first part g(T) represents the Arrhenius equation meant for the dependence of reaction rate on temperature.

(11) $$D=g\left(T\right)\exp\left\{\left(a_{0}T-a_{1}\right)M\right\}$$

(12) $$g\left(T\right)=D_{0}exp\left(-\frac{E_{a}}{RT}\right)$$

where $a_{0}$ and $a_{1}$ are the unknown coefficients, $D_{0}$ is pre-exponential factor (m^2^.s^−1^), $E_{a}$ is the energy of activation (kJ.mol^−1^), and R is the universal gas constant (8314.3 J mol^−1^.K^−1^).

### 2.8 Initial and boundary Conditions

The following initial conditions were considered:

1. Uniform initial temperature of lentil seeds T_0_ having a range between 3 and 6 °C for different treatments.
2. Uniform initial moisture content of sample M_0_ according to the amount of tempering for each treatment.

The boundary conditions for heat transfer Equation (2) at the bottom and top sample surface can be expressed by Equation (13) (Jiang et al., 2017).

(13) $$\vec{n}.\left(k\nabla T\right)=h\left(T_{a}-T\right)$$

Here, $\vec{n}$ is the outward unit normal vector, h is the convective heat transfer coefficient (W.m^−2^.K^−1^), $T_{a}$ is the ambient air temperature considered an average of 296.15 K during the treatment.

The boundary conditions for solving mass transfer Equation (9) can be obtained by equation (14) (Hemis et al., 2012).

(14) $$\vec{n}.\left(-D_{eff}\nabla M\right)=h_{m}\left(M-M_{e}\right)$$

Here, $\;h_{m}$ is the convective mass transfer coefficient (m.s^−1^), $M_{e}$ is the equilibrium moisture content considered to be zero (Heydari et al., 2020).

By using the definition of Biot number, the magnitudes of the convective mass and heat transfer were estimated. The dimensionless Biot number correlates the internal heat conduction or mass diffusion in solids to the external convection (Giner et al., 2010). The graphs, which were firstly published by Gurney and Lurie (1923), and obtained by analytical solving the unsteady state heat conduction equation with an infinite series for several Biot numbers with the inherent assumption that the Biot number stays constant during the process, have been used by many chemical and food engineers. In this study, the optimum values for the heat and mass transfer Biot numbers which are calculated by Equations (15), (16) (Yıldız et al., 2007), were obtained through the optimization algorithm, so the convective heat and mass transfer coefficients were determined based on that.

(15) $$Bi_{C}=\;\frac{hL}{K}$$

(16) $$Bi_{m}=\;\frac{h_{m}L}{D}$$

where $Bi_{C}$ and $Bi_{m}$ are the heat and mass transfer Biot number, and L is the characteristic length calculated by dividing the volume to the surface area of the seed and found to be 0.48 (mm).

### 2.9 Simulation procedure

The system of Equations (2), (9) is a nonlinear set of partial differential equations (PDE) with Neumann boundary conditions Equations (13), (14), and there is no analytical solution for such a system and can only be solved using numerical methods. Among different numerical methods, the finite element method was employed. The model was solved by developing code in MATLAB (Version 9.7.0.1190202, R2019b, The MathWorks Inc, Natick, MA, USA) and using its PDE Toolbox. The geometry and meshing of lentil seed with 506 nodes and 1069 triangle elements have been shown in the Graphical abstract.

### 2.10 Optimization algorithm to estimate unknown coefficients

Moisture content and surface temperature are two factors that are measured experimentally during the process and can also be predicted by solving a mathematical model. Therefore, two sum square errors for the predicted and experimental measurements can be calculated, and the best coefficients for Equations (10), (11), (12) can be estimated by minimizing these errors. This means we are facing a multiobjective optimization problem, and to find the solution for this system, the FGOALATTAIN solver in Optimization Toolbox™ of MATLAB software has been employed. The function finds the minimum of the problem specified as follow:

(17) $$\begin{array}{l} minimize\;\gamma \;such\;that\\ CM,\gamma \end{array}\left\{ \begin{array}{l} F\left(CM\right)-weight\times \gamma \leq goal\\ Loss\;factor\;values>0\\ lb\;\leq C\leq ub \end{array}\right.$$

where γ is the parameter that software tries to minimize and CM is the unknown coefficient matrix for the estimated loss factor, and effective moisture diffusivity equations, weight matrix considered as [1, 1] to make the problem unscaled goal attainment, lb and ub are the lower and upper bounds respectively in the algorithm specified in the range compatible with the values reported by other researchers (Husain et al., 1973; Sipahioglu and Barringer, 2003; Taheri et al., 2018; Tang and Sokhansanj, 1993).

The goal and initial point are the values needed to be specified for solving the above optimization problem and making the process converge to a feasible point. For this aim, another step has been considered to find out appropriate initial guess points as well as goal values. In this step, it was tried to optimize each of the sum square errors separately and proportional to their directing impact factors. In other words, the sum square error of surface temperature (SSET_S_) was minimized by the optimization of the coefficients of loss factor equation used in the heat transfer equation, and the minimum of the sum square error of moisture content (SSEMC) was obtained by optimizing the effective moisture diffusivity equation's coefficient applied in mass transfer equation. However, the loss factor and effective moisture diffusivity and the other parameters in the model are affected by temperature or moisture content, or both. This means that an accurate estimation of both affecting factors is needed to obtain accurate results. It has been assumed that the whole seed has uniform moisture content and temperature equal to the measured values during the process and was modeled by using five terms of Fourier Series according to the time of process for each treatment. In this way, instead of solving a coupled model, the attempt will be focused only on solving the heat or mass transfer equation and finding the coefficients of loss factor and effective moisture diffusivity as the initial values for the FGOALATTAIN algorithm. FMINSEARCH function in MATLAB, with an interior-point algorithm (Byrd et al., 1999; Waltz et al., 2006), was employed to find the optimal guess points in this step. FGOALATTAIN optimization algorithm stops when the search direction size becomes less than twice the value of the step size tolerance (1e-6) while the constraints are satisfied. The recorded history of the multiobjective algorithm iterations for different treatments has been provided in Appendix A, Figures A1-4. The record includes the search directions to create optimum new points and the corresponding optimum objective functions in each iteration until converging to a feasible point.

### 2.11 Thermal properties

After the thermal process by microwave, the lentil seeds were milled using an impact miller (NutriMill, SKU – 760200, NutriMill®, Utah, USA), equipped with a high-speed impact chamber stainless steel milling heads to create fine flour. Then the thermal properties of obtained flours were evaluated by using a differential scanning calorimeter (Model Q 2000 TA Instruments, New Castle, DE, USA) calibrated with indium according to the method of Setia et al. (2019) with some modification. Lentil flour (3.3–3.6 mg) was precisely weighed into a hermetic aluminum pan (TA Instruments, New Castle, DE, USA), then deionized water (∼3–4 volumes, v/w) was added prior to sealed hermetically and allowed to equilibrate at ambient temperature overnight. The sealed pans were heated from 10 to 180 °C at a heating rate of 10 °C per minute. The thermal properties onset (To), peak (Tp), and conclusion (Tc) temperatures, as well as enthalpy changes (ΔH), were calculated from the observed endothermic peak in the DSC thermogram with the use of the Universal Analysis 2000 Software (TA Instruments, New Castle, DE, USA).

### 2.12 Fourier transform mid-infrared (FT-MIR) spectroscopy

The FT-MIR spectroscopy of the raw and microwave-treated lentil samples was carried out at the mid-IR beamline of the Canadian Light Source Inc., Saskatoon, SK. The FT-MIR data were collected using an Agilent Technologies (Cary 670 series, Agilent Technologies Inc., CA, USA) microscope equipped with a bulk analysis accessory and thermoelectrically cooled Deuterated Lanthanum α-Alanine doped TriGlycine Sulphate (DLaTGS) detector at the mid-IR beamline at the Canadian Light Source, Saskatoon, Canada. Lentil flour samples were prepared for FT-MIR in the form of pressed potassium bromide (KBr) pellets, with sample material representing 1.1–1.3% of the total weight of the KBr pellets. 4.7–5.1 mg of sample were cryogrinding and homogenization with 399.3–401.4 mg of KBr using a Spex SamplePrep Geno/Grinder 2010. Three replicates for each sample mixed KBr with a 97.4–99.0 mg weight were measured for each sample. KBr pellet was pressed with a 13 mm utilizing an automated hydraulic press (AutoCrushIR PIKE Technologies Inc., Madison, WI, USA). The process of pellet preparation and data collection for FT-MIR was based on the method described by Karunakaran et al. (2020). The FT-MIR data analysis was performed using Quasar (Toplak et al., 2017) software (version 0.5.6) (10.5281/zenodo.4287478). To study starch structure alterations during the process, the FT-MIR spectra were truncated to 1200-900 cm^−1^, normalized based on the sample weights in KBr Pellet, then baseline corrected using rubber band correction algorithm, and finally vector normalized.

## 3 Results and discussion

### 3.1 The predicted parameters of the loss factor equation

**Fig. 1:**
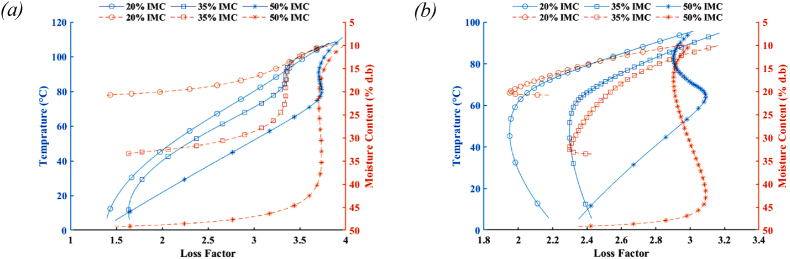
Variation of loss factor according to the changes in the mean temperature and moisture content of lentil seeds tempered to various moisture contents processing in (a) microwave at 0.7 kW nominal power and (b) microwave at 0.35 kW nominal power.

The optimization algorithm for the heat and mass transfer equations model of microwave treatments of tempered lentil seeds resulted in the range of coefficients obtained for the loss factor equation provided in Table 1. By looking at the values, the slight difference between the same coefficients under different treatments of microwave powers and tempering amounts can be observed. By considering the mean values of moisture content and temperature for the lentil seed during the thermal treatment, the average changes in loss factor can be estimated in the process, illustrated in Fig. 1. Marker indices in this figure and similar figures have been used to show the changes in related parameters every 15 s during the microwave thermal treatment, which can provide a view of the variation rate in related factors. The range of loss factor variations is between 1.39 to 3.97 and 1.95 to 3.17 for the lentil seeds treatment in the microwave at 0.7 kW and 0.35 kW nominal power, respectively. The reported values for this property for red lentils having moisture content between 8.6 and 19% at room temperature are between 0.25 and 1.8, increasing by the rise in moisture content (Taheri et al., 2018).

**Table 1:** The optimal coefficients obtained for the loss factor equation base on the thermal treatment of lentil seed at different microwave powers and initial moisture contents.

Treatment MW Power/IMC	g_0_	g_1_	g_2_	g_3_	g_4_	g_5_	g_6_	g_7_
0.70 kW/20% (d.b)	2.100	−0.111	2.81E-04	−1.37E-03	1.00E-03	2.966	−1.670	0.0461
0.70 kW/35% (d.b)	2.100	−0.100	4.62E-04	−1.00E-02	9.34E-04	2.964	−1.674	0.0329
0.70 kW/50% (d.b)	2.119	−0.040	5.07E-05	−1.99E-02	4.58E-04	2.853	−1.709	0.0300
0.35 kW/20% (d.b)	2.100	−0.151	1.01E-04	−1.00E-02	1.00E-05	2.686	−1.264	0.0700
0.35 kW/35% (d.b)	2.160	−0.112	5.00E-05	−1.00E-04	5.93E-05	2.820	−1.476	0.0580
0.35 kW/50% (d.b)	2.161	−0.100	6.02E-05	−1.00E-02	5.88E-04	2.964	−1.602	0.0506
Average	2.123	−0.102	1.68E-04	−8.56E-03	5.08E-04	2.876	−1.566	0.0479
Standard Deviation	0.030	0.036	1.69E-04	7.17E-03	4.20E-04	0.113	0.169	0.0151

**Fig. 2:**
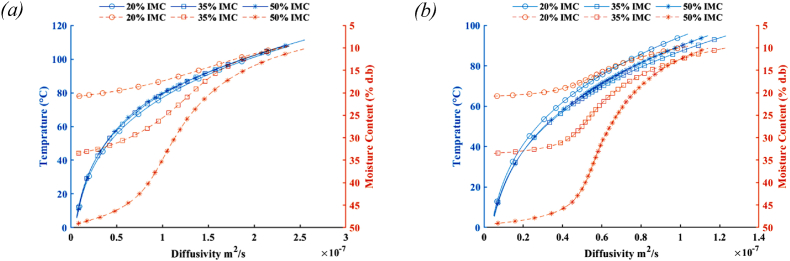
Variation of diffusivity according to the changes in the mean temperature and moisture content of lentil seeds tempered to various moisture contents processing in (a) microwave at 0.7 kW nominal power and (b) microwave at 0.35 kW nominal power.

**Fig. 3:**
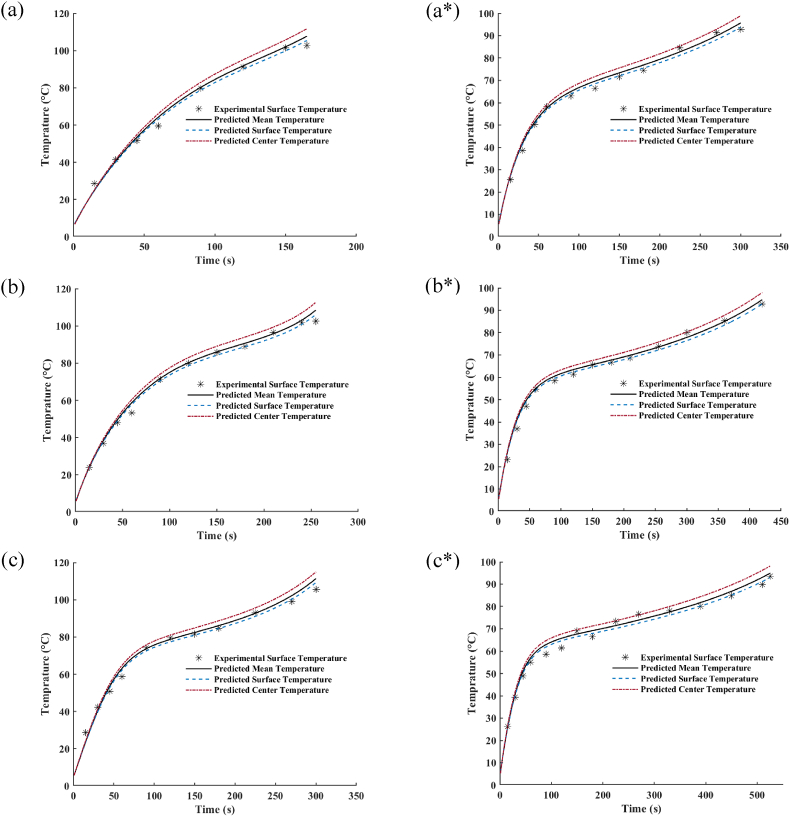
Variation in the experimental surface temperature and the prediction of mean, surface and center temperature changes of lentil seed tempered to various moisture contents (a) tempered to 20%; (b) tempered to 35%; (c) tempered to 50%, processing in microwave at 0.7 kW nominal power on the left side, and at 0.35 kW nominal power on the right side indicating by * superscript.

**Fig. 4:**
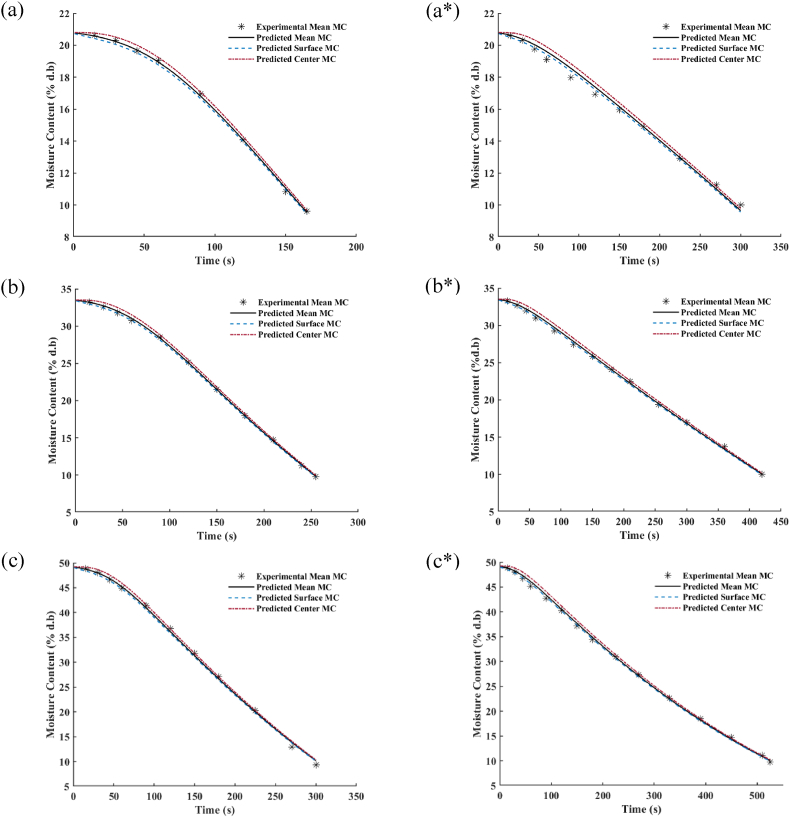
Variation in the experimental mean moisture content and the prediction of mean, surface and center moisture content changes of lentil seed tempered to various moisture contents (a) tempered to 20%; (b) tempered to 35%; (c) tempered to 50%, processing in microwave at 0.7 kW nominal power on the left side, and at 0.35 kW nominal power on the right side indicating by * superscript.

During the microwave process, by raising the temperature of seeds while their moisture content is reducing, for samples with initial moisture contents of 20 and 35%, the loss factor shows a growth trend. This behavior can be explained by the loss factor property dependency to its consisting parts: ionic and dipole loss (Sipahioglu and Barringer, 2003). Increasing temperature not only leads to the rise in ionic conduction due to the lowering viscosity (Wang et al., 2013) but also causes an increased dipole loss behavior because of the availability of more free water molecules (Taheri et al., 2018).

Regarding the behavior of loss factor during the microwave process for the samples tempered to the highest moisture content, 50%, it can be observed that loss factor firstly increases until reaching the transition point at temperatures about 81 and 64 °C for the microwave powers of 0.7 and 0.35 kW, respectively, then it starts to decrease for a while until returns to the growth behavior. Since starch is the major component of lentils, consisting of around 41–46% of its chemical composition (Wang and Daun, 2006), this variation in loss factor can be attributed to the starch gelatinization happening during the process.

Gelatinization occurs when starch is heated in the presence of surplus water at a specific temperature range called gelatinization temperature. During this thermal process condition, starch granules start irreversibly swelling and undergoing an order-to-disorder phase transition and become amorphous (Ai and Jane, 2015; Cooke and Gidley, 1992). Starch gelatinization also restricts the mobility of dipoles water molecules leading to the reduction of their ability to align with the alternating electric field (Motwani et al., 2007). However, the mixing of small water polar molecules with long-chain starch will enhance its indistinct polarization ability (Tao et al., 2020). For these reasons, the behavior of the seed's loss factor is not beyond the expectation, although the starch concentration is another key factor (Motwani et al., 2007) resulted in observing such a trend for the samples tempered to the highest moisture content.

### 3.2 The predicted parameters of effective moisture diffusivity equation

The range of coefficient for effective moisture diffusivity equation calculated by using the mentioned optimization algorithm has been illustrated in Table 2. In the present study, the activation energy values for lentils during the microwave process are discovered to be in the domain of 29–30 kJ.mol^−1^ lying in the range of 12.7–110 kJ.mol^−1^ for drying agricultural and food materials (Khanali et al., 2016). The mean variations in lentil diffusivity during microwave treatment can be predicted by considering the average values of moisture content and temperature of seeds in the process, shown in Fig. 2. Temperature is the decisive factor in determining the effective moisture diffusivity in a way that by increasing temperature during the process, although the reduction in moisture content is observed, the effective moisture diffusivity is rising in a growth trend. In the current study, the estimated domain variations of effective moisture diffusivity were obtained to be between 4.46 × 10^−9^ to 2.55 × 10^−7^ m^2^^.^s^−1^, which is in the range of 2.06 × 10^−14^ to 1.07 × 10^−6^ m^2^.s^−1^ found in the literature for legumes (Panagiotou et al., 2004).

**Table 2:** The optimal coefficients obtained for the effective moisture diffusivity equation base on the thermal treatment of lentil seed at different microwave powers and initial moisture contents.

Treatment MW Power/IMC	D_0_	E_a_	a_0_	a_1_
0.70 kW/20% (d.b)	3.56E-03	30.315	1.00E-06	0.397
0.70 kW/35% (d.b)	3.46E-03	30.314	1.00E-06	0.397
0.70 kW/50% (d.b)	2.36E-03	29.026	1.00E-06	0.500
0.35 kW/20% (d.b)	1.32E-03	29.000	1.00E-03	0.300
0.35 kW/35% (d.b)	2.45E-03	30.300	1.00E-03	0.300
0.35 kW/50% (d.b)	1.48E-03	29.011	1.00E-03	0.300
Average	2.44E-03	29.66	5.01E-04	0.366
Standard Deviation	9.45E-04	0.71	5.47E-04	0.081

### 3.3 The optimum values of Biot numbers

The optimum values for heat transfer Biot number obtained by optimization algorithm were the same for all treatments except for the sample tempered to the 50% moisture content and processed by microwave powers of 0.7 kW having slightly lower values, which were equal to 0.043 and 0.038, respectively. For mass transfer Biot numbers, the same values of 0.015 were found for all treatments with initial moisture contents of 20 and 35%. This value for the samples tempered to the highest moisture content of 50% was slightly higher and equal to 0.016. Generally, it has been recognized that for the heat and mass transfer Biot number below 0.1, the surface resistance throughout the surrounding medium of the open boundary layer is considerably greater than the internal resistance to the heat conduction and mass diffusion within the solid body (Górnicki et al., 2019; Ramaswamy et al., 1982). These low Biot number values also confirm the finding of other researchers about the accumulation of moisture content at food surface during the microwave process because of driving more moisture content to the surface that can be removed by surrounding cooler air (Datta and Ni, 2002).

### 3.4 Experimental and simulated results for temperature

The experimental surface temperature and its prediction, as well as simulated mean and center temperature changes of lentil seed under the different microwave powers treatments, have been illustrated in Fig. 3 to show how the algorithm in the study predicts the temperature variation during the microwave process of tempered lentil seeds by estimating loss factor and diffusion model through the minimizing SSET_s_ and SSEMC. Generally, the rise in the microwave power leads to the increase of lentil seeds final mean temperature, which the maximum mean temperature of 111 °C have been obtained for lentils with the initial moisture content of 50% and processed under microwave powers of 0.7 kW and the minimum amount of 95 °C found for the seeds processed under microwave power of 0.35 kW. For the lentils tempered to 20 and 35% moisture content, the final average temperatures of samples when processed under 0.7 kW microwave power were about 107 and 109 °C, respectively, which is lower than the sample under this treatment with the initial moisture content of 50% (Contour plot of temperature distribution inside the processed lentil seed at the end of the microwave thermal treatment has been provided in Appendix B, Figure B1).

The minimum values obtained for SSET_s_ through the optimization process, as well as the statistical performance indicators of the simulated model for different treatments, have been given in Table 3. It was found that SSET_s_, the coefficient of determination (R^2^), root mean square error (RMSE), and the mean relative percentage error (MRPE) of the model ranges from 39.985 to 79.972, 0.9922 to 0.9958, 1.8257 to 2.5815, and 140.4423 to 207.4533, respectively. The high values for R^2^ and low values of RMSE confirm the accuracy of the model in forecasting temperature variation of tempered lentil seed during the microwave process.

**Table 3:** The statistical performance indicators of the simulated model output for the mean surface temperature variation in the thermal treatment of lentil seed at different microwave powers and initial moisture contents.

Treatment MW Power/IMC	SSET_S_a)	R^2^	RMSE	MRPE
0.70 kW/20% (d.b)	39.9852	0.9957	2.1078	174.7507
0.70 kW/35% (d.b)	48.2606	0.9958	2.0054	148.8306
0.70 kW/50% (d.b)	79.9720	0.9922	2.5815	207.4533
0.35 kW/20% (d.b)	39.9991	0.9950	1.8257	148.2306
0.35 kW/35% (d.b)	49.9924	0.9935	1.8897	140.4423
0.35 kW/50% (d.b)	66.9593	0.9923	2.0457	164.1014
Average	54.1948	0.9941	2.0760	163.9681
Standard Deviation	16.0231	0.0016	0.2683	24.6476

a SSET_S_: Sum Square Error of Surface Temperature; R^2^: Coefficient of Determination; RMSE: Root Mean Square Error; MRPE: Mean Relative Percentage Error.

### 3.5 Experimental and simulated results for moisture content

The variations in the experimental mean moisture content and its estimated values, as well as simulated surface and center moisture content changes of lentil seed under the different microwave powers process, have been illustrated in Fig. 4. When the microwave treatment starts, the gradient in moisture content inside the seed appears, and the difference in moisture content between the surface and center of the sample begins to increase. However, as the processing time passes, it can be found that this difference is reduced in a way that at the end of the process, the seed approximately has uniform moisture content distribution (Contour plot of moisture content distribution inside the processed lentil seed at the end of the microwave thermal treatment has been provided in Appendix B, Figure B2). This confirms the fact that the increase of effective moisture diffusivity because of the rise in temperature during the microwave process results in uniform moisture inside the seed.

The minimum values achieved for SSEMC through the optimization algorithm, as well as the statistical performance indicators of the simulated model for different treatments, have been provided in Table 4. It was found that SSEMC, the coefficient of determination (R^2^), root mean square error (RMSE), and the mean relative percentage error (MRPE) of the model have ranged from 1.02E-5 to 2.96E-4, 0.9929 to 0.9997, 1.06E-3 to 4.96E-3, and 0.082 to 0.401, respectively. These values also confirm the precise estimation of this model system for moisture content variation.

**Table 4:** The statistical performance indicators of the simulated model output for the mean moisture content variation in the thermal treatment of lentil seed at different microwave powers and initial moisture contents.

Treatment MW Power/IMC	SSEMCa)	R^2^	RMSE	MRPE
0.70 kW/20% (d.b)	1.02E-05	0.9993	1.06E-03	0.0820
0.70 kW/35% (d.b)	2.32E-05	0.9997	1.39E-03	0.1043
0.70 kW/50% (d.b)	2.96E-04	0.9987	4.96E-03	0.4008
0.35 kW/20% (d.b)	1.09E-04	0.9929	3.01E-03	0.2305
0.35 kW/35% (d.b)	8.79E-05	0.9988	2.51E-03	0.1962
0.35 kW/50% (d.b)	2.37E-04	0.9992	3.85E-03	0.3144
Average	1.27E-04	0.9981	2.80E-03	0.2214
Standard Deviation	1.16E-04	0.0026	1.48E-03	0.1222

a SSEMC: Sum Square Error of Moisture Content; R^2^: Coefficient of Determination; RMSE: Root Mean Square Error; MRPE: Mean Relative Percentage Error.

### 3.6 Thermal Properties

**Fig. 5:**
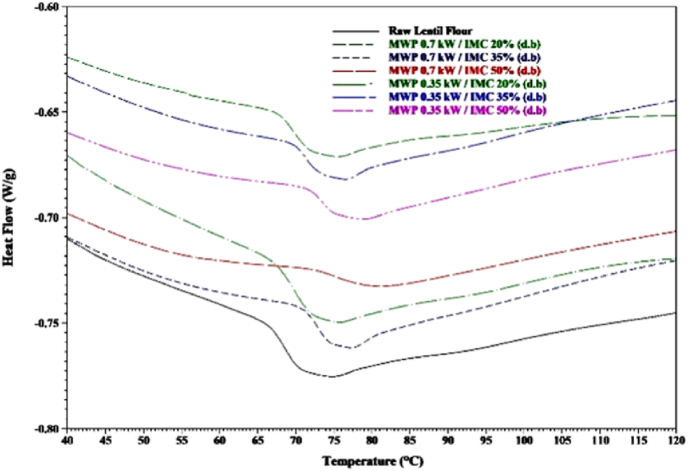
DSC thermograms of the raw and microwave-treated lentil flours.

The DSC thermograms of the raw lentil flour and flour obtained from the microwave process of the tempered lentil seeds exhibited one endothermic peak, and microwave processing resulted in the shift of these peaks to higher temperatures (Fig. 5). More details of these thermal transition peaks have been provided in Table 5, and it can be found that raw lentil flour has the lowest peak temperature of 70.90 °C, and the highest value, 81.03 °C is for the flour of lentil seeds tempered to 50% moisture content and thermally processed at 0.70 kW microwave power. The data also reveals that the microwave treatment of lentil seeds with the initial moisture content of 20% will not significantly increase the values of thermal transition peaks compared to the values of raw lentil flour. Villanueva et al. (2018) also reported that the microwave treatment of wet rice flour would raise the starch gelatinization temperature and showed that this is because of the higher crystallites' perfection leading to the need for greater gelatinization temperatures to melt starch crystallites. In addition, the gelatinization enthalpy of flour from microwave-treated tempered seed reduces compared to the raw flour, and the highest significant reduction was observed for the treatments with the highest initial moisture content of 50%, showing the partial starch gelatinization occurred in the samples due to the microwave process (Lewandowicz et al., 2000; Villanueva et al., 2018). This is also confirming the fact that the occurrence of much more starch gelatinization for these samples leads to the appearance of transition points in their loss factor trends during the microwave process.

**Table 5:** Thermal properties and crystallinity of the raw lentil flours and the flours obtained from the thermally processed lentil seed at different microwave powers and initial moisture contents.

Treatment MW Power/IMC	To (°C)a)	Tp (°C)	Tc (°C)	ΔH (J/g)	DO
Raw lentil flour	67.45^e^	70.90^d^	78.70^d^	4.103^a^	1.024^a^
0.70 kW/20% (d.b)	68.06^e^	71.89^d^	80.43^dc^	2.278^cba^	1.021^a^
0.70 kW/35% (d.b)	71.31^c^	74.65^c^	81.62^cb^	1.566^dcb^	1.014^b^
0.70 kW/50% (d.b)	73.80^a^	81.03^a^	89.62^a^	0.246^d^	0.988^d^
0.35 kW/20% (d.b)	67.94^e^	71.95^d^	79.70^dc^	3.258^ba^	1.023^a^
0.35 kW/35% (d.b)	70.54^d^	73.71^c^	81.73^cb^	2.352^cba^	1.017^b^
0.35 kW/50% (d.b)	72.25^b^	76.82^b^	83.44^b^	0.932^dc^	0.998^c^

In the same column, the values with the same letter are not significantly different at P < 0.05.

a T_o_: onset temperature; T_p_: peak temperature; T_c_: conclusion temperature; and ΔH: enthalpy change; DO: degree of order.

### 3.7 FT-MIR spectroscopy

**Fig. 6:**
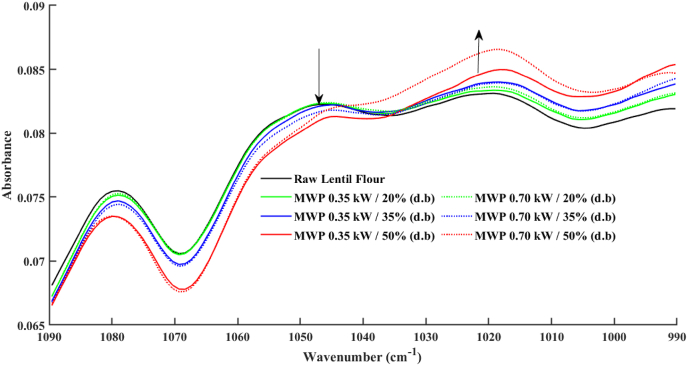
The average and normalized FT-MIR spectra of the raw and microwave-treated lentil flours.

The spectra of raw and microwave-treated lentils are shown in Fig. 6. The spectra absorptions bands in the 970–1090 cm^−1^ have been selected because of the sensitivity of starch gelation in this band (Rubens et al., 1999). The effect of microwave thermal treatment on the starch structure of lentil samples can be observed through the variation in absorption intensity at specific bands. The ratio of IR bands at 1017 and 1047 cm^−1^ are responsive to the amount of amorphous and crystalline region in starch structure, respectively (Rubens et al., 1999). Spectra in Fig. 6 shows a minor increase of 1017 cm^−1^ for 20% moisture compared to raw. Processing at higher moisture contents, 35% and 50%, further increases the IR band at 1017 cm^−1^ while decreasing 1047 cm^−1^ and therefore increasing amorphous starch. Divekar et al. (2017) also reported higher and sharper FT-MIR normalized absorbance for microwave processed pulses flours, including lentils, compared to the raw samples. The internal changes in the degree of order (DO) or crystallinity of starch in samples can be estimated and evaluated by the ratio of their FT-MIR relative intensities at 1047/1017 cm^−1^ (Alsalman and Ramaswamy, 2021; Chávez-Murillo et al., 2018; Ma et al., 2018; Xu et al., 2019). The results reveal that the DO values of the lentil flours obtained from the microwave processed grains tempered to 35% and 50% reduced significantly compared to the raw lentil flour, having the value of 1.024 (Table 5). The sharp reductions were observed for the thermally processed samples with the highest initial moisture content of 50%, which proves the importance of initial moisture content during starch gelatinization and shows the higher starch structure alteration for these samples, causing the occurrence of transition point in their loss factor trends during the microwave process. In addition, it can be observed that using higher microwave power (0.7 kW) for thermal processing of these samples (tempered to 50%) led to the more significant decline in the DO values of modified flours, which can be attributed to higher temperature reaching of seeds under this treatment. These observations are in agreement with DSC results. Ma et al. (2018) reported reducing the DO values for the microwave and autoclaved thermal treatment of isolated lentil resistant starch. The study on the structural changes in starch from untreated and autoclaved chickpea, navy bean, and yellow field pea seeds, done by Xu et al. (2019), also proves that thermal treatment reduces crystallinity, leading to the reduction in the DO values of processed starch.

## 4 Conclusions

The optimization algorithm developed in this study can successfully estimate the parameters of the loss factor and moisture diffusivity models for the lentil seed during the microwave process. The interesting finding of this research is that the estimated model for the loss factor shows the occurrence of transition point in this property for the seeds tempered to the highest moisture content, 50%, showing the effect of starch gelatinization on this property. The significantly higher peak temperatures and noticeably lower gelatinization enthalpies and degree of order in the DSC thermograms and the FT-MIR spectroscopy of the flours obtained from these processed seeds also confirmed the greater intensity of starch structure alteration happened in these samples, affecting the dielectric loss factor property. Therefore, the proposed method for modeling the loss factor and moisture diffusivity is a promising procedure not only to cover the wide range of affecting factors, moisture content, and temperature but also to illustrate how the chemical reaction occurring during the microwave process influences the loss factor property which determines the amount of microwave energy absorption. These findings will aid in the suitable design and scale-up consideration for building microwave applicators.

## Recommendation for future study

The findings of this study should be seen considering some limitations. Although all precautions were considered to reduce the sources of errors, there are some potential errors associated with the uncertainty of assumptions as well as off-line measurements of the sample's weight loss and surface temperature. Therefore, the experiment design with the capability of online or real-time recording for the mentioned factors not only can reduce the potential source of errors but also provide a large reliable data source improving the accuracy of the predicted models.

## CRediT authorship contribution statement

**Mohamad Mehdi Heydari:** Conceptualization, Data curation, Formal analysis, and, Investigation, Writing – original draft, Writing - review & editing. **Tahereh Najib:** Writing - review & editing. **Oon-Doo Baik:** Writing - review & editing. **Kaiyang Tu:** Writing - review & editing, FTIR setup and data collection. **Venkatesh Meda:** Conceptualization, Data curation, Formal analysis, and, Investigation, Writing - review & editing.

## Declaration of competing interest

The authors declare that they have no known competing financial interests or personal relationships that could have appeared to influence the work reported in this paper.

## Data Availability

The data that support the findings of this study can be made available by the corresponding author upon reasonable request.
