# Supplementary material for: Loss factor and moisture diffusivity property estimation of lentil crop during microwave processing
Source: Curr Res Food Sci. 2021 Dec 25;5:73–83. doi: 10.1016/j.crfs.2021.12.008 (PMC8724939; doi:10.1016/j.crfs.2021.12.008)

**Appendix A**


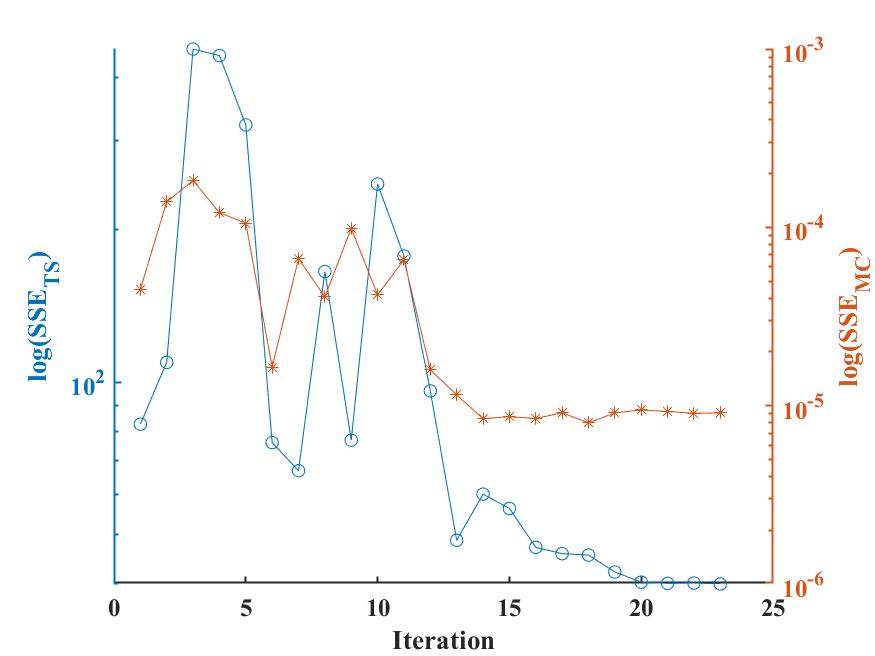

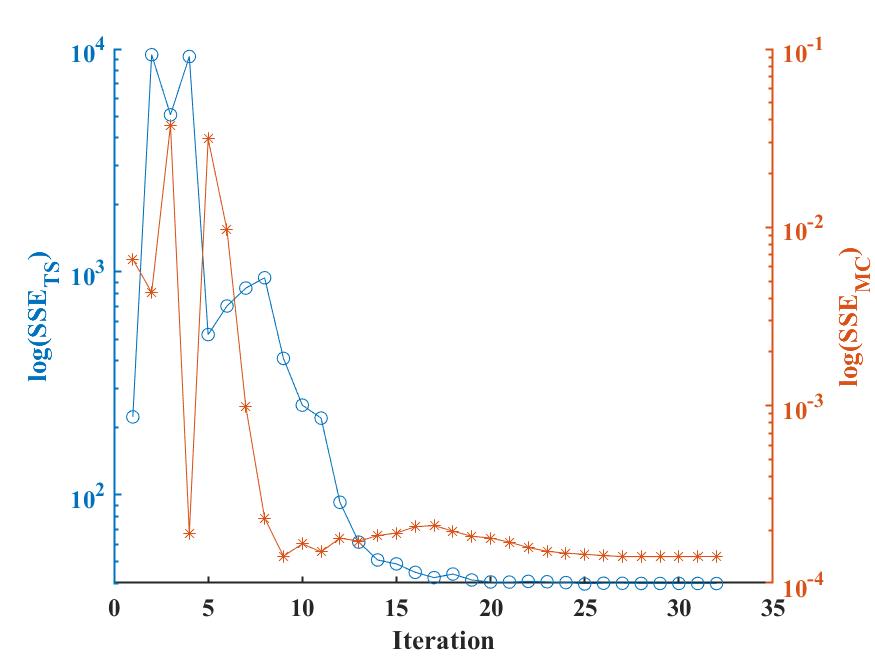


(a)


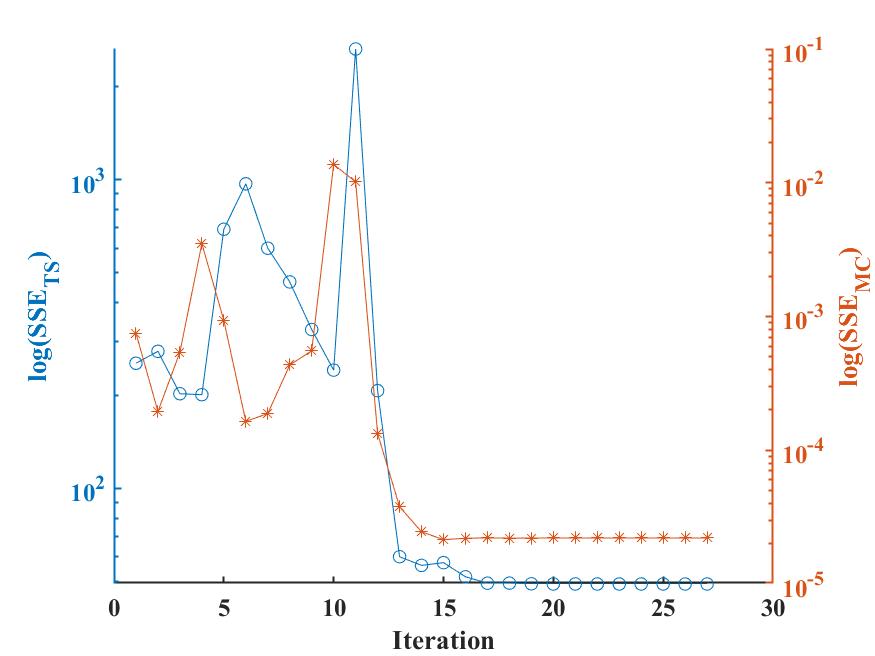


(b)

(a^*^)


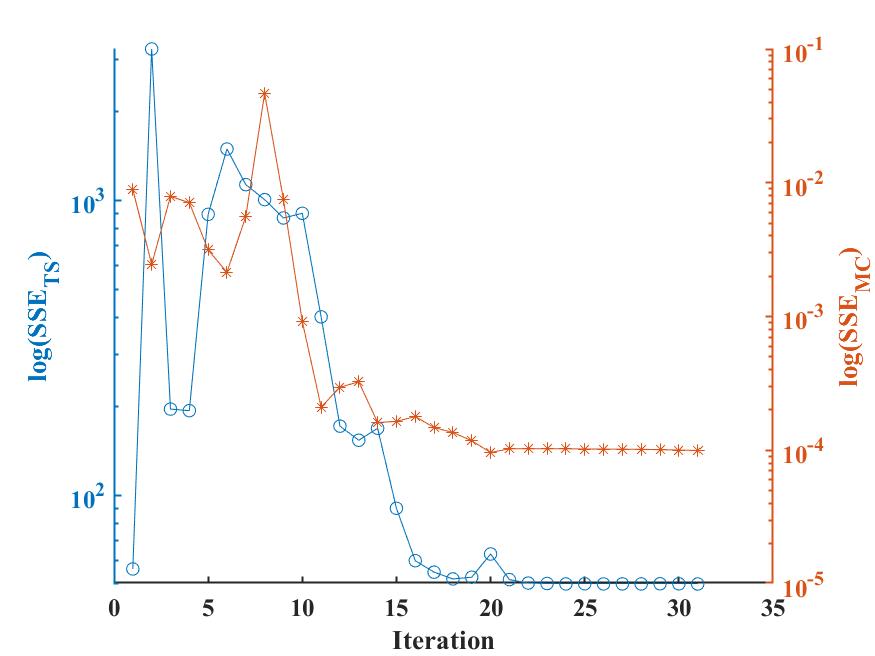

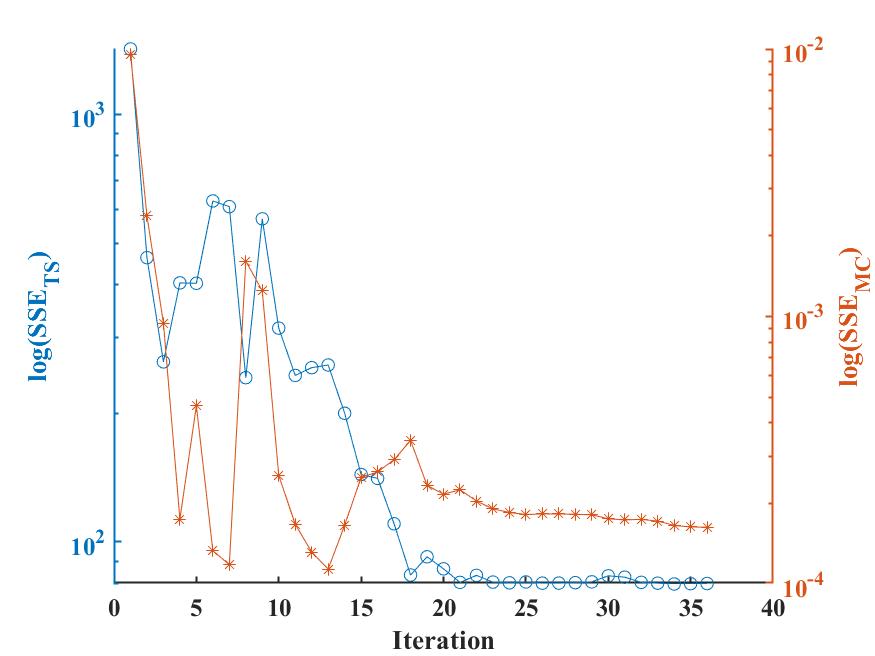

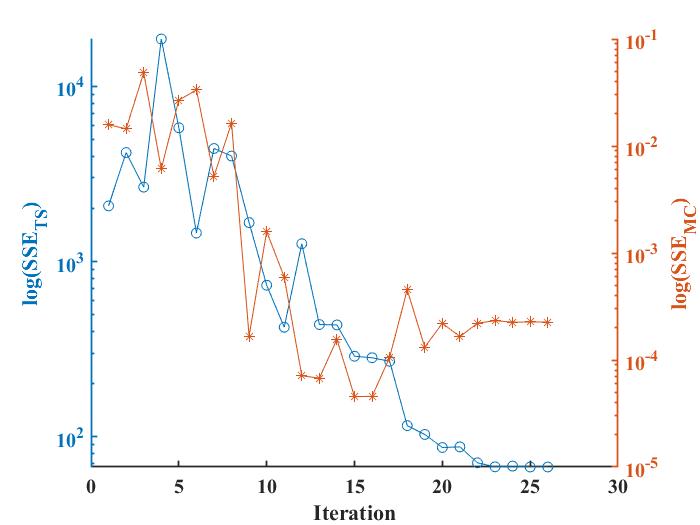


(b^*^)

(c)

(c^*^)

Figure A.1 Reduction in the SSE_TS_ and SSE_MC_ during the running of the multiobjective algorithm for the lentil seed tempered to various moisture contents (a) tempered to 20 %; (b) tempered to 35%; (c) tempered to 50%, processing in microwave at 0.7 kW nominal power on the left side, and at 0.35 kW nominal power on the right side indicating by ^*^ superscript. Reaching to the feasible point happened after 25 to 35 iterations for various treatments (the number of function evaluations (model runs) were different, having the range between 500 to 600. It also needs to consider that the number of model evaluations to find the initial point was in the field of 3000 to 5000). The axes for sum square errors are in log form in this figure because of the different scales for SSE_TS_ and SSE_MC_ and providing a better view by covering a wide range of variations.


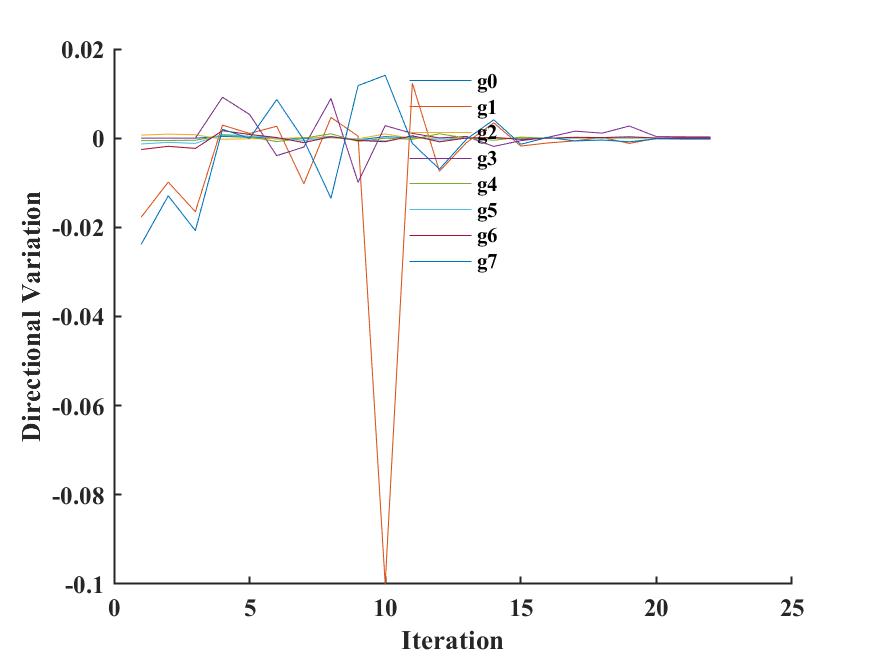

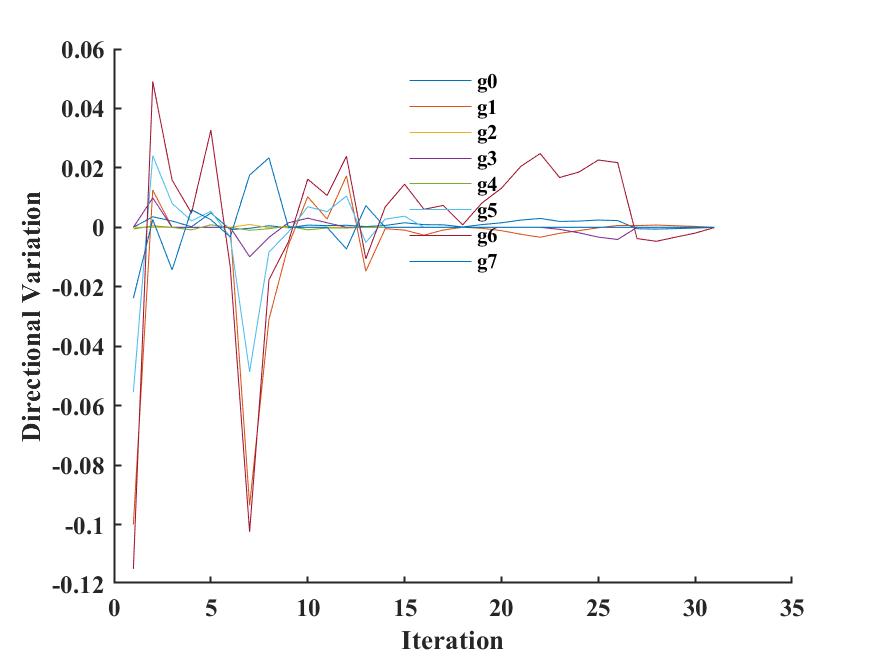


(a)


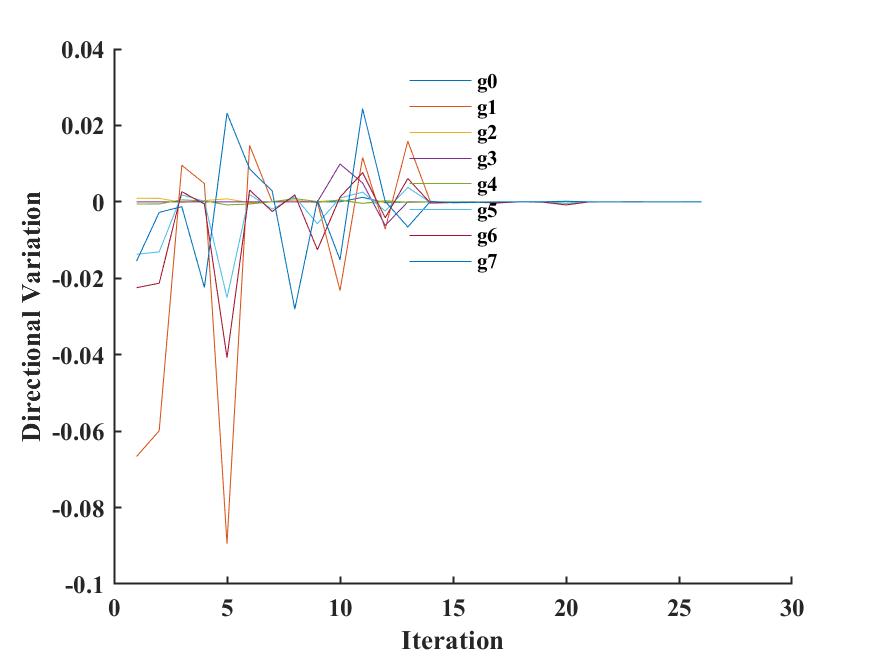


(b)

(a^*^)


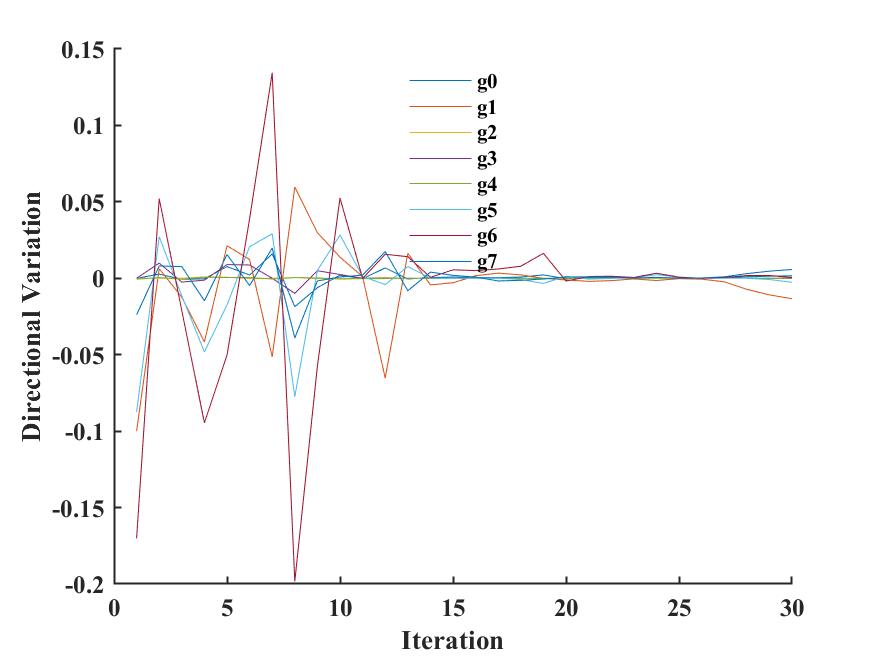

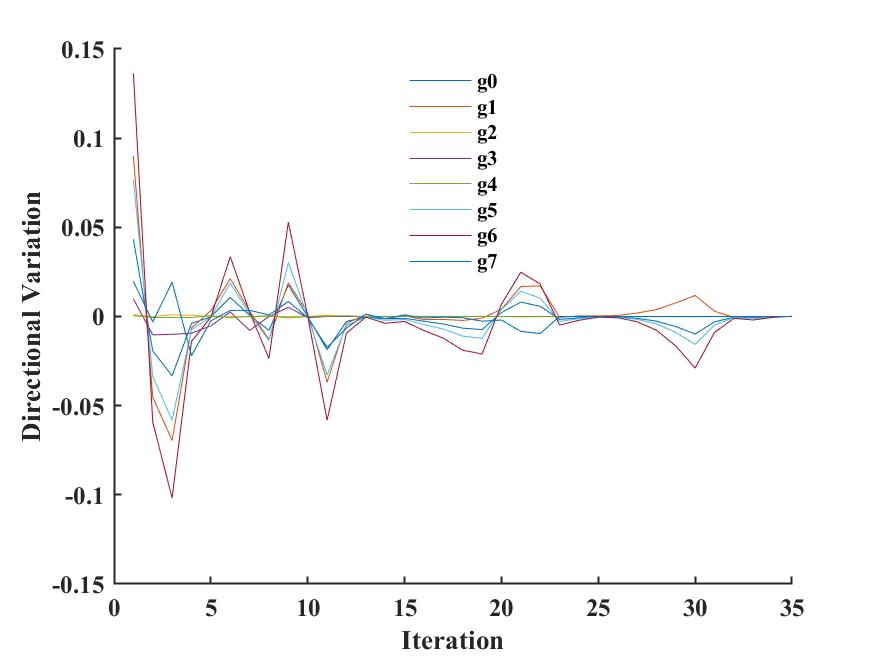

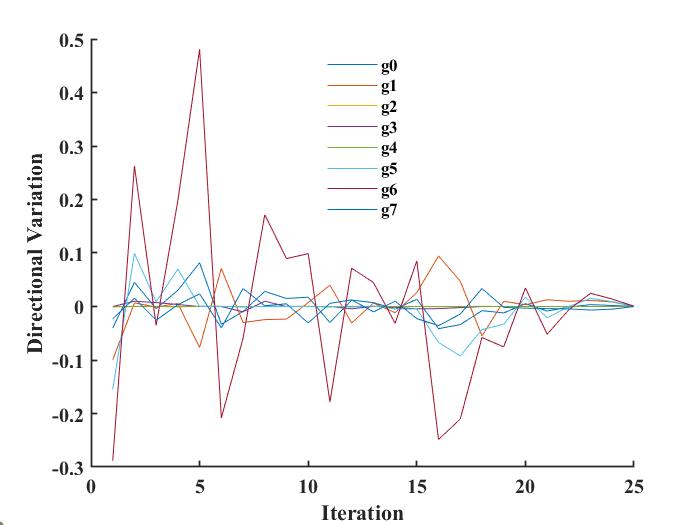


(b^*^)

(c)

(c^*^)

Figure A.2 Directional changes in the coefficients of loss factor equation during the running of the multiobjective algorithm for the lentil seed tempered to various moisture contents (a) tempered to 20 %; (b) tempered to 35%; (c) tempered to 50%, processing in microwave at 0.7 kW nominal power on the left side, and at 0.35 kW nominal power on the right side indicating by ^*^ superscript.


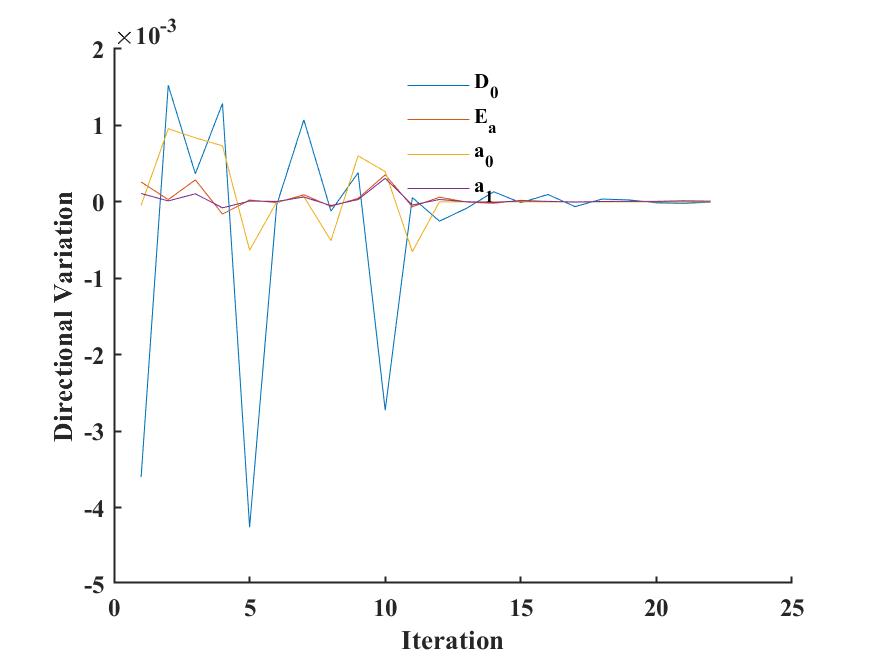

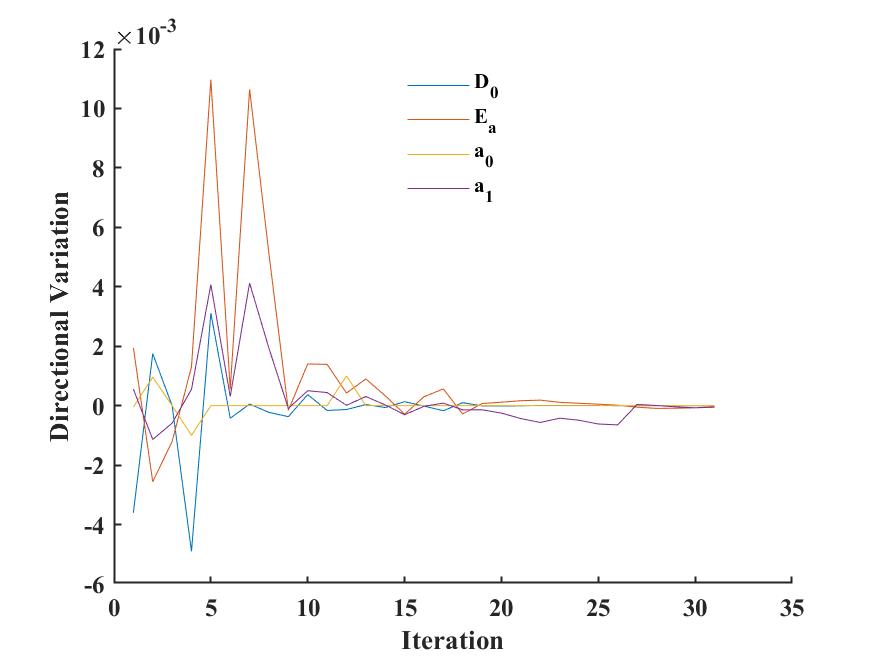


(a)


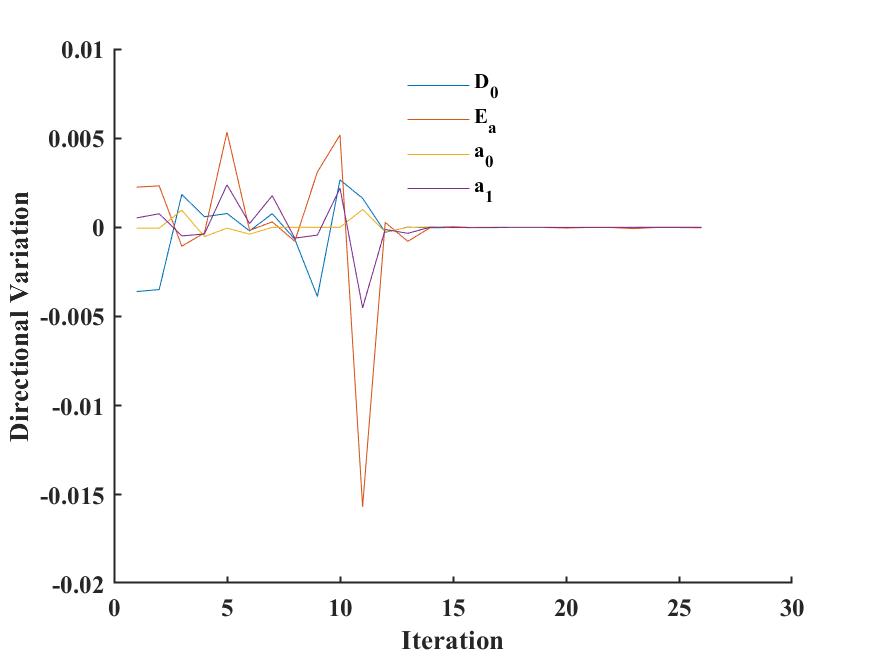


(b)

(a^*^)


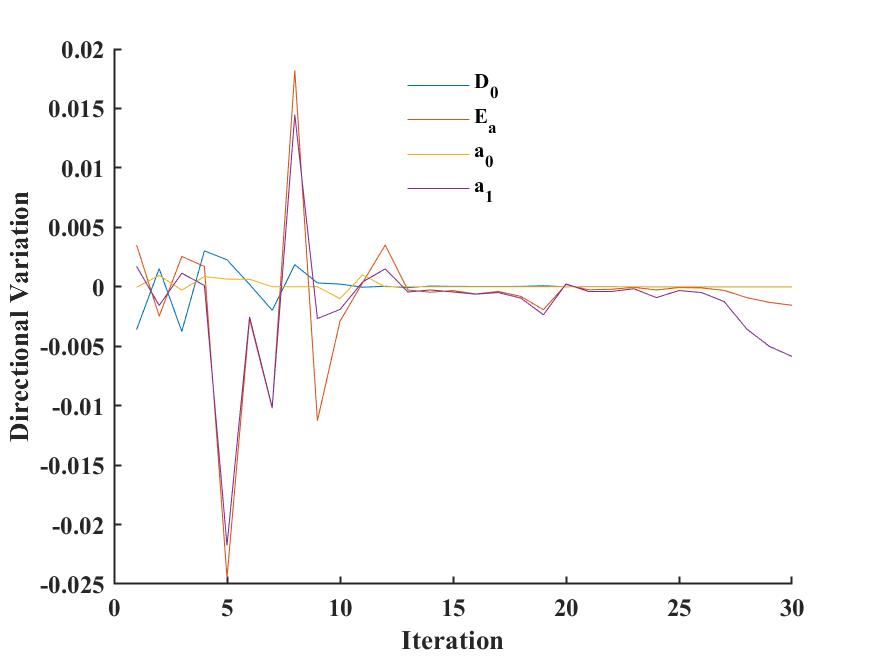

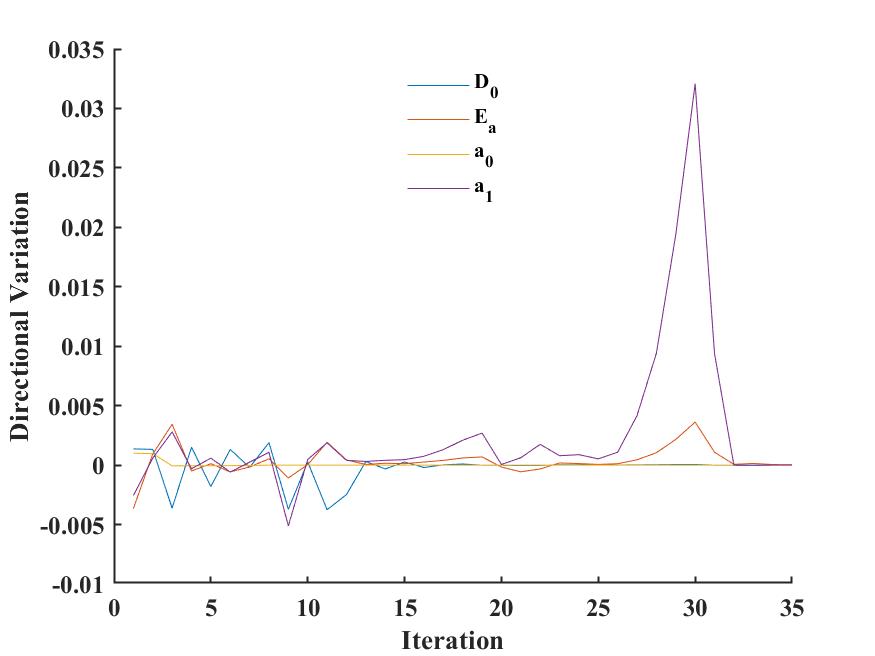

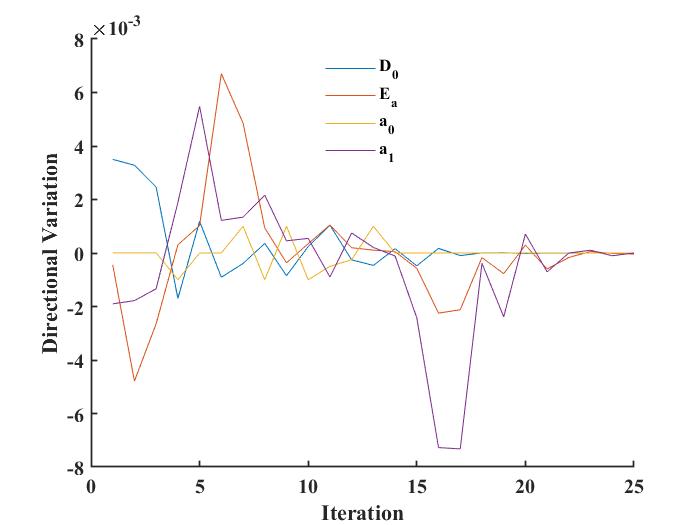


(b^*^)

(c)

(c^*^)

Figure A.3 Directional changes in the coefficients of moisture diffusivity equation during the running of the multiobjective algorithm for the lentil seed tempered to various moisture contents (a) tempered to 20 %; (b) tempered to 35%; (c) tempered to 50%, processing in microwave at 0.7 kW nominal power on the left side, and at 0.35 kW nominal power on the right side indicating by ^*^ superscript.

s


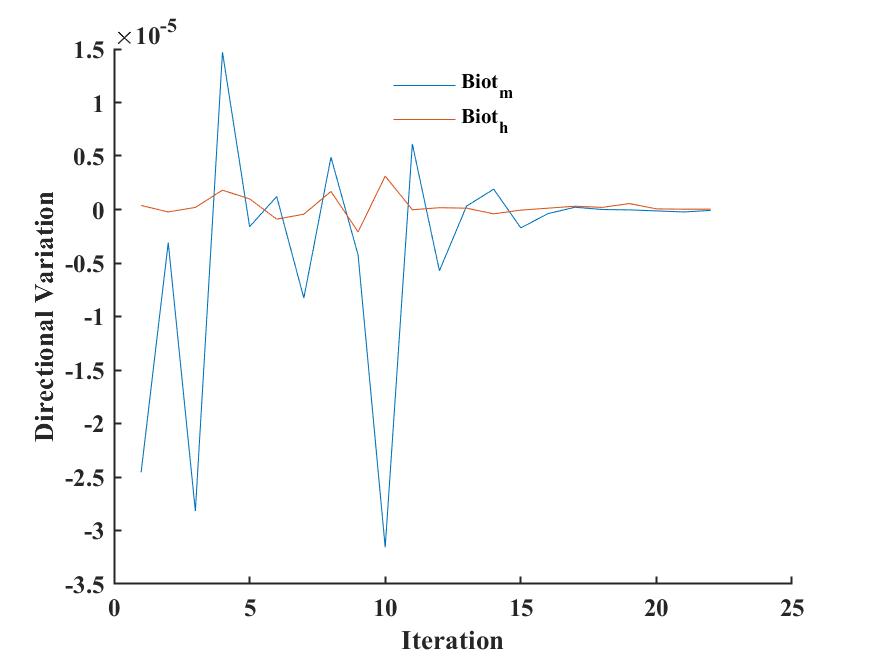

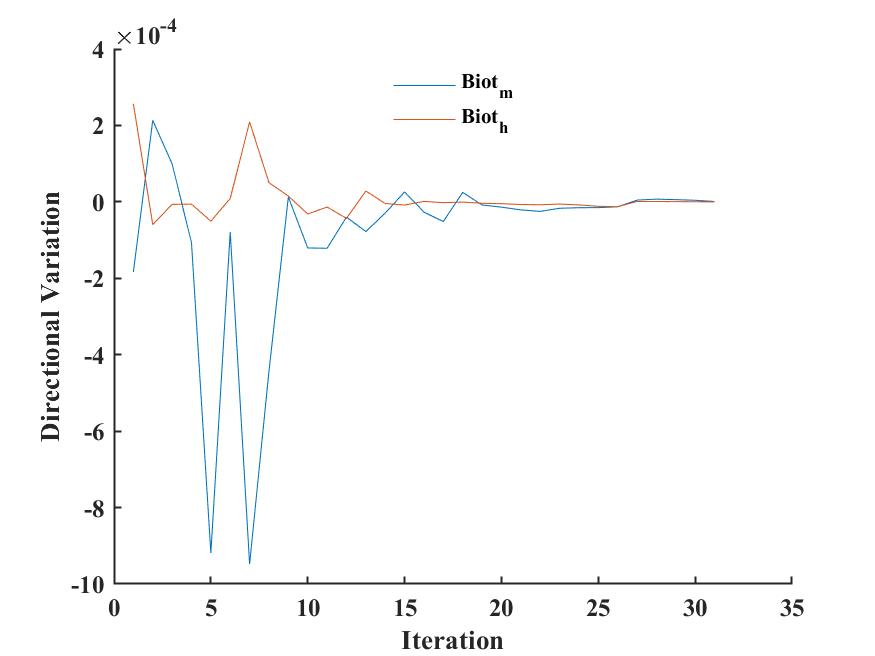


(a)


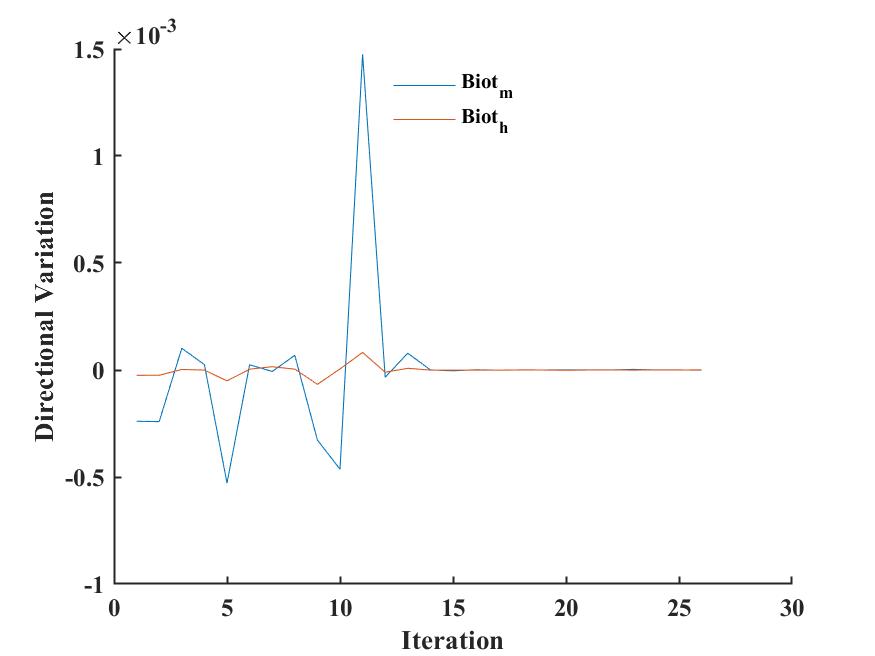


(b)

(a^*^)


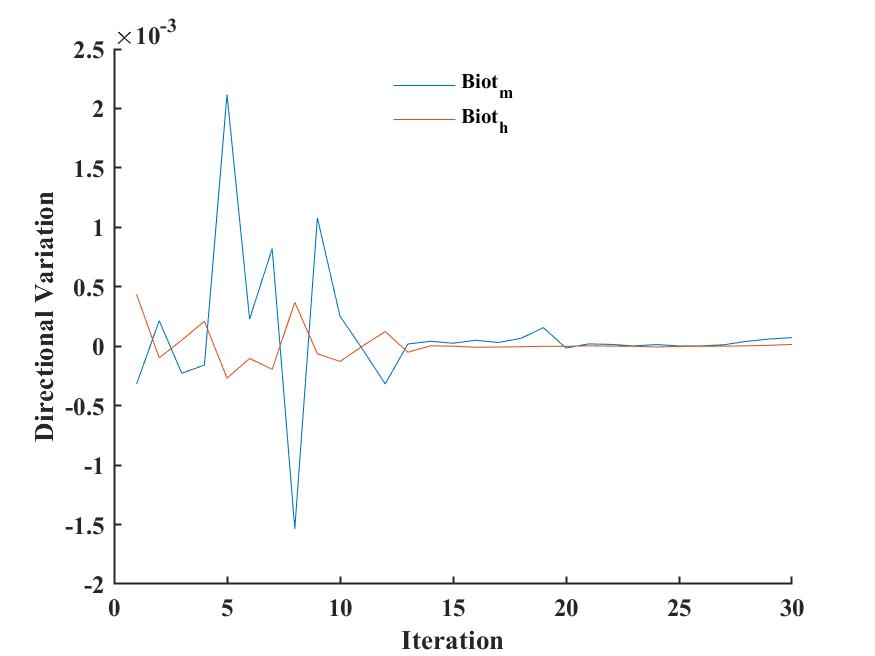

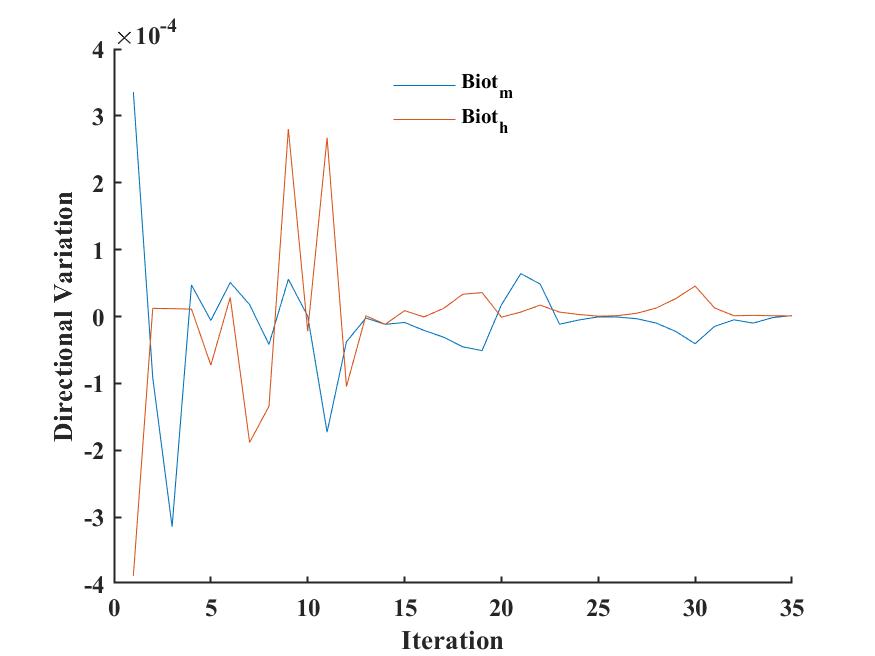

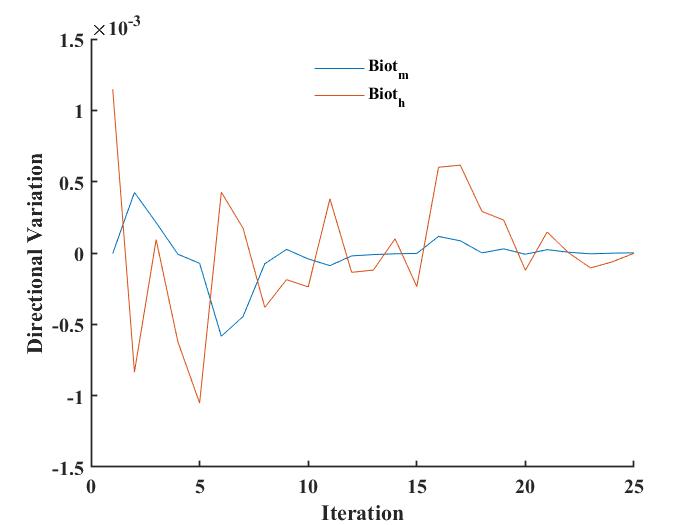


(b^*^)

(c)

(c^*^)

Figure A.4 Directional changes in the heat and mass transfer Biot number during the running of the multiobjective algorithm for the lentil seed tempered to various moisture contents (a) tempered to 20 %; (b) tempered to 35%; (c) tempered to 50%, processing in microwave at 0.7 kW nominal power on the left side, and at 0.35 kW nominal power on the right side indicating by ^*^ superscript.

**Appendix B**

Figure B.1 Contour plot of temperature distribution inside the processed lentil seed at the end of the microwave thermal treatment of the seed tempered to various moisture contents (a) tempered to 20 %; (b) tempered to 35%; (c) tempered to 50%, at 0.7 kW microwave nominal power on the left side, and at 0.35 kW microwave nominal power on the right side indicating by ^*^ superscript.


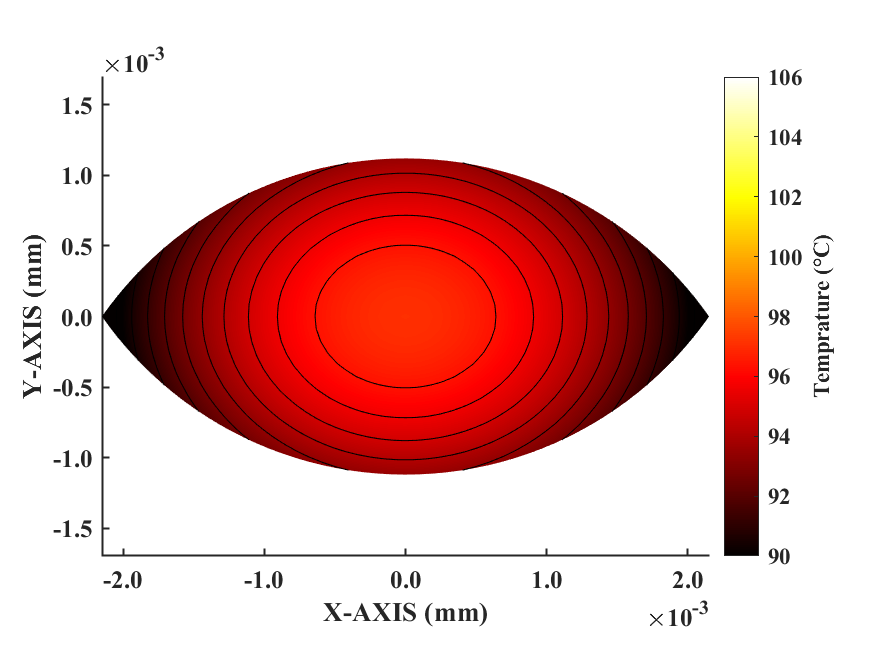


(a)

(b)

(a*)


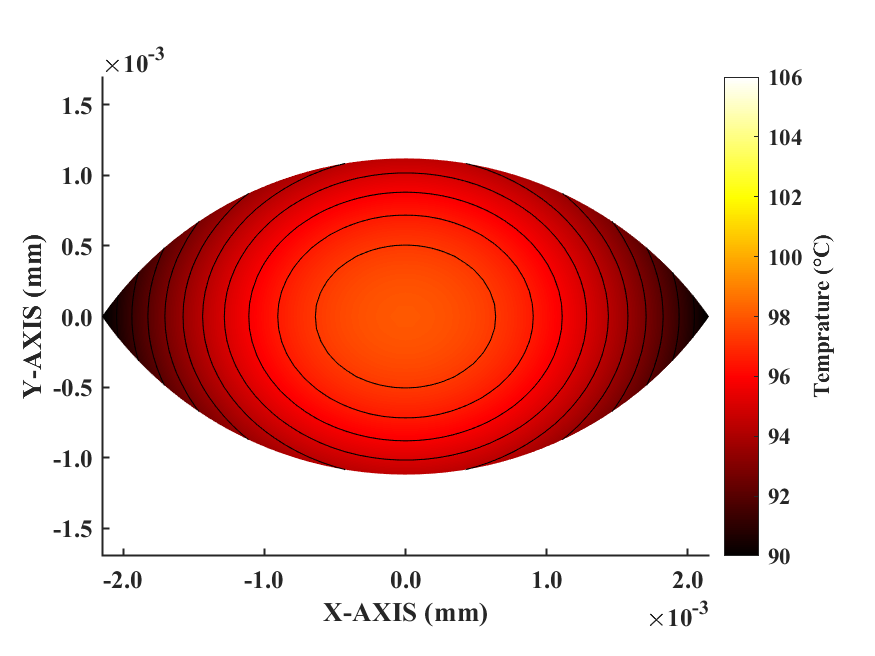

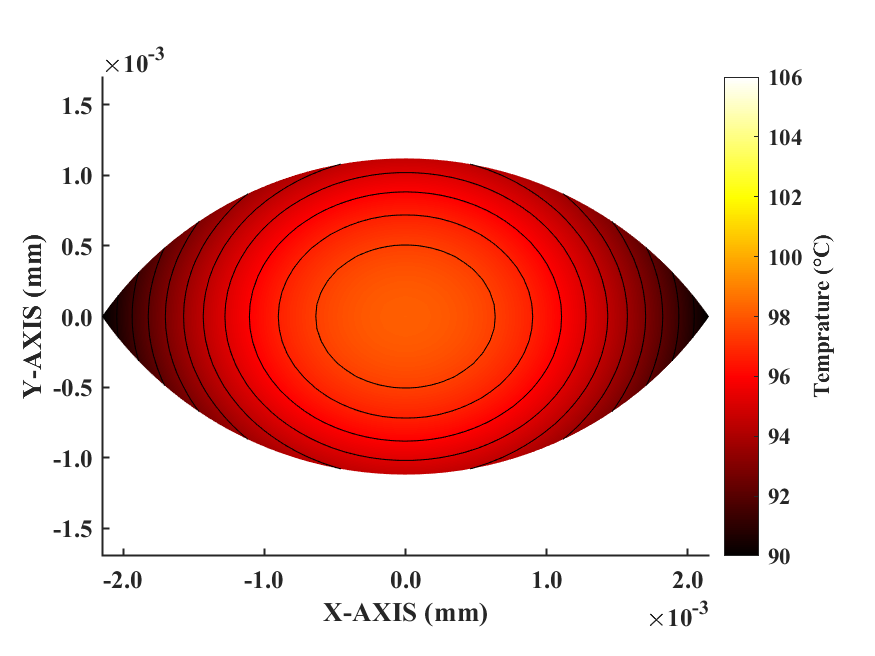


(b*)

(c)

(c*)


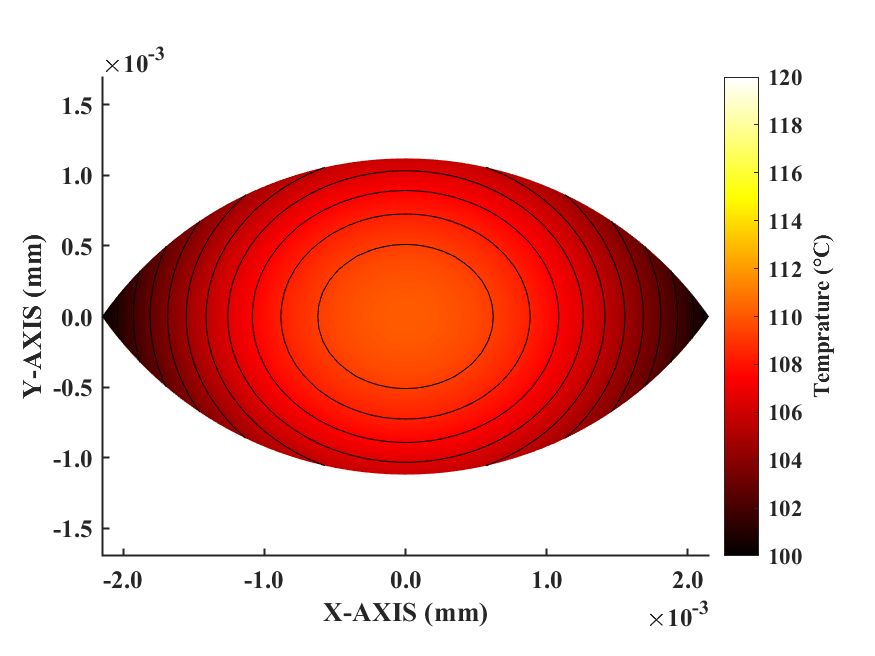

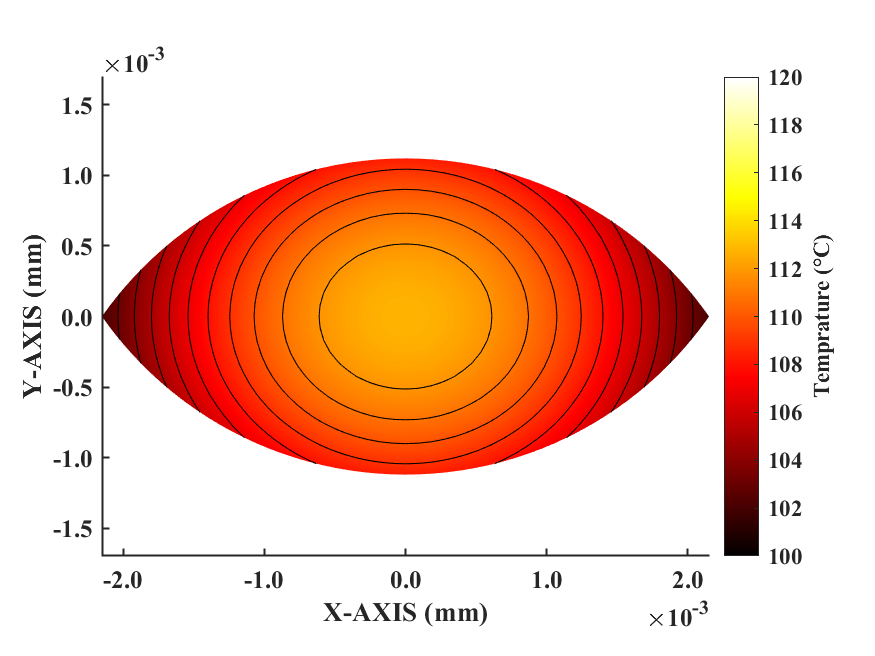

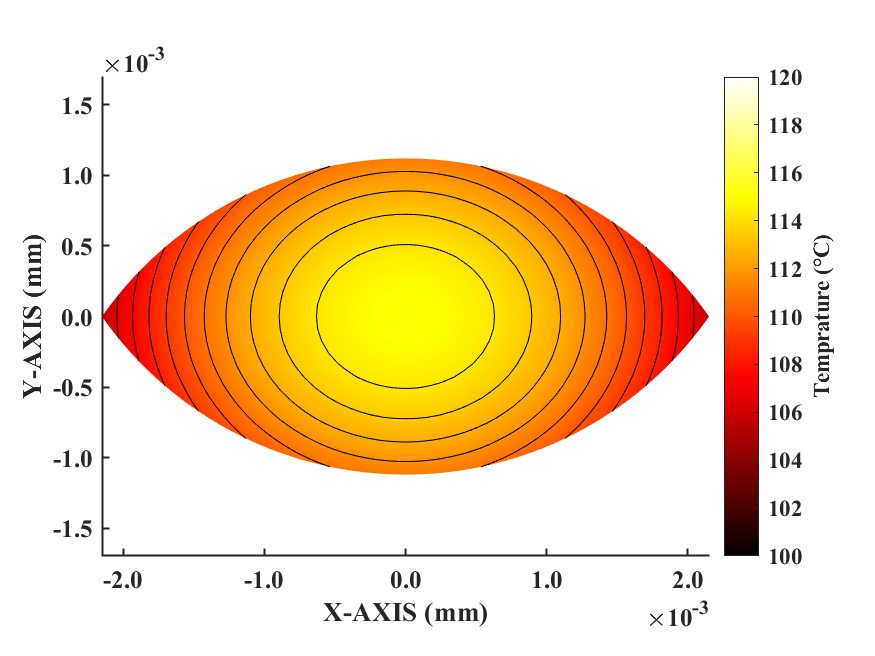


Figure B.2 Contour plot of moisture content inside the processed lentil seed at the end of the microwave thermal treatment of the seed tempered to various moisture contents (a) tempered to 20 %; (b) tempered to 35%; (c) tempered to 50%, at 0.7 kW microwave nominal power on the left side, and at 0.35 kW microwave nominal power on the right side indicating by ^*^ superscript.


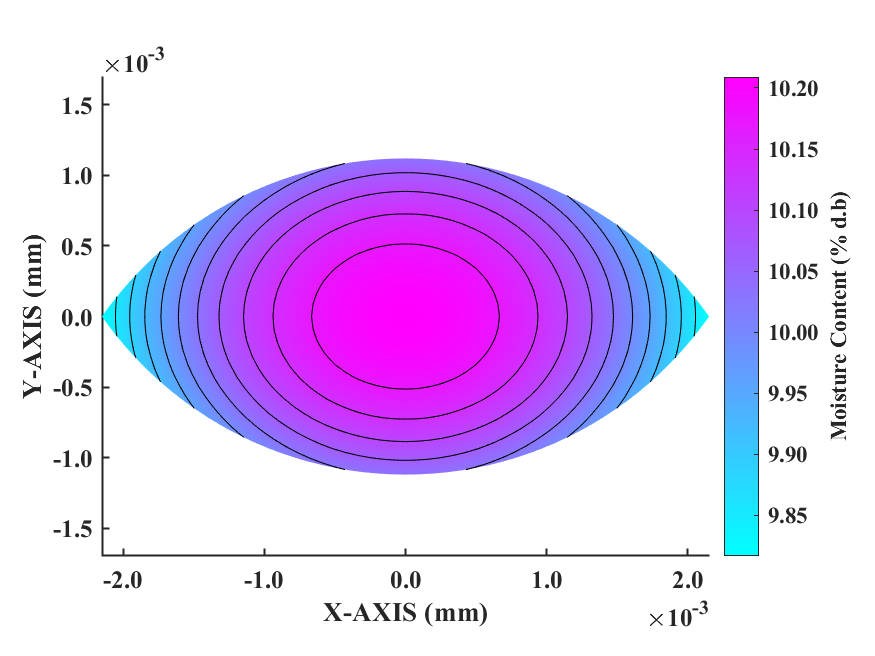


(a)

(b)

(a*)


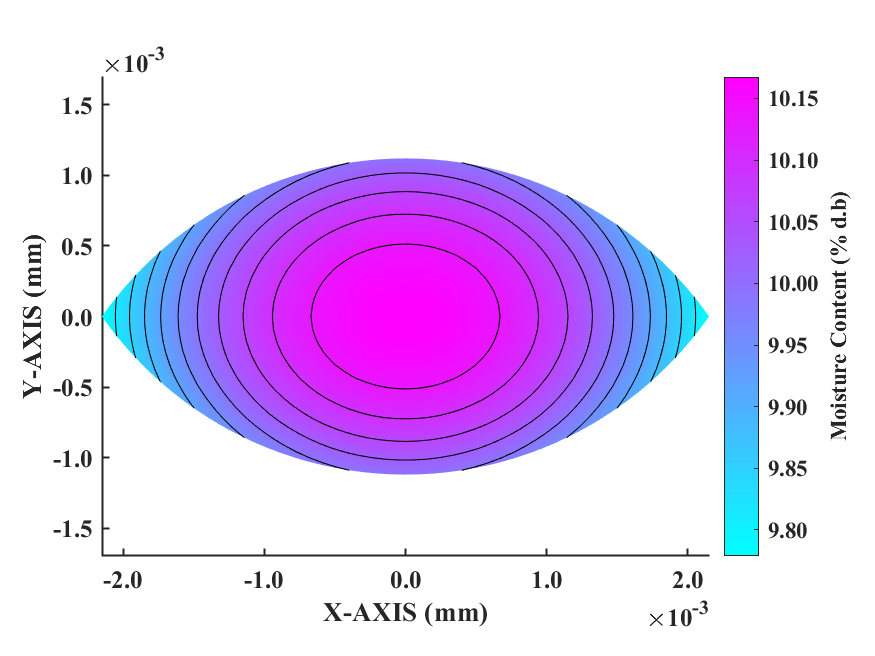

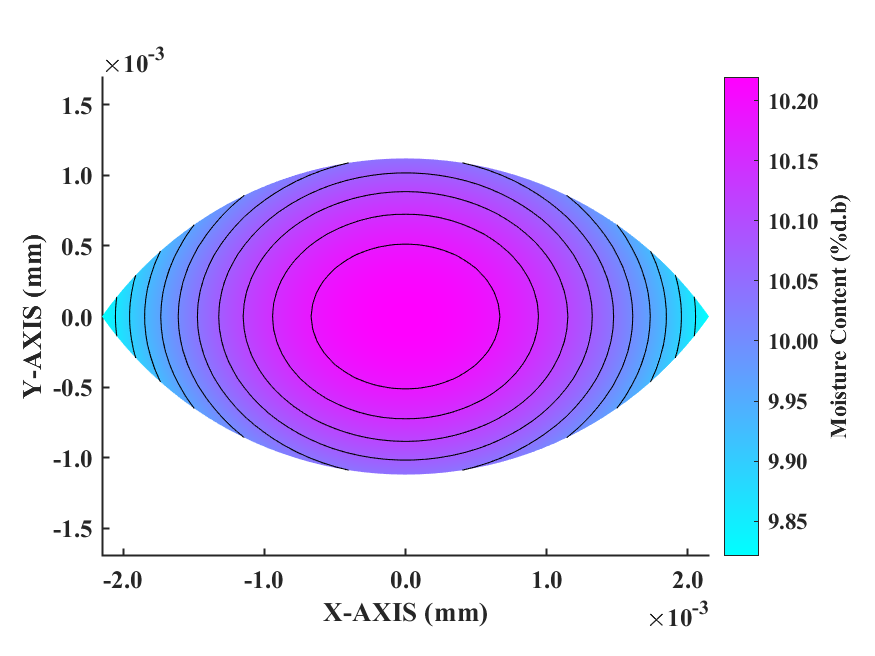


(b*)

(c)

(c*)


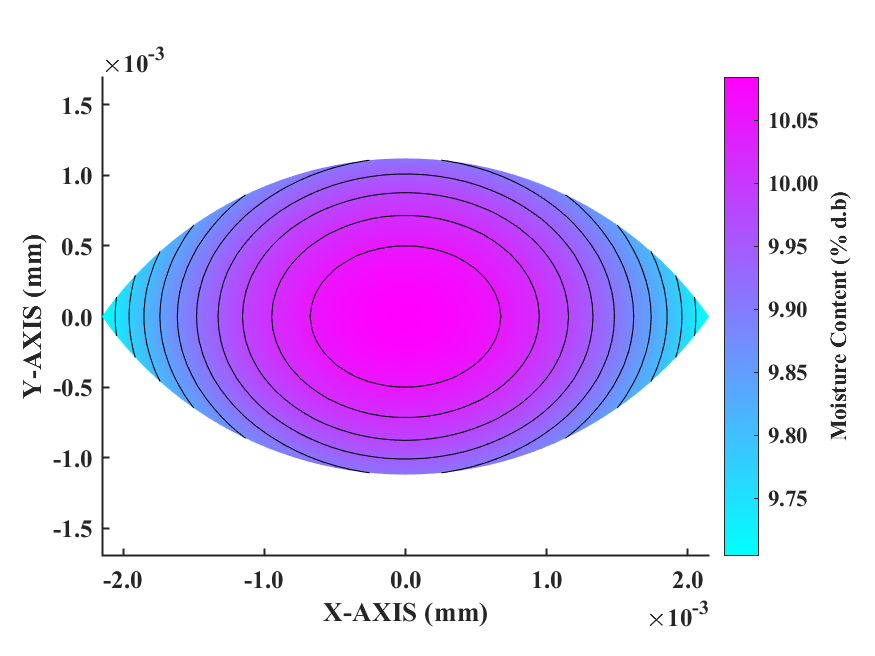

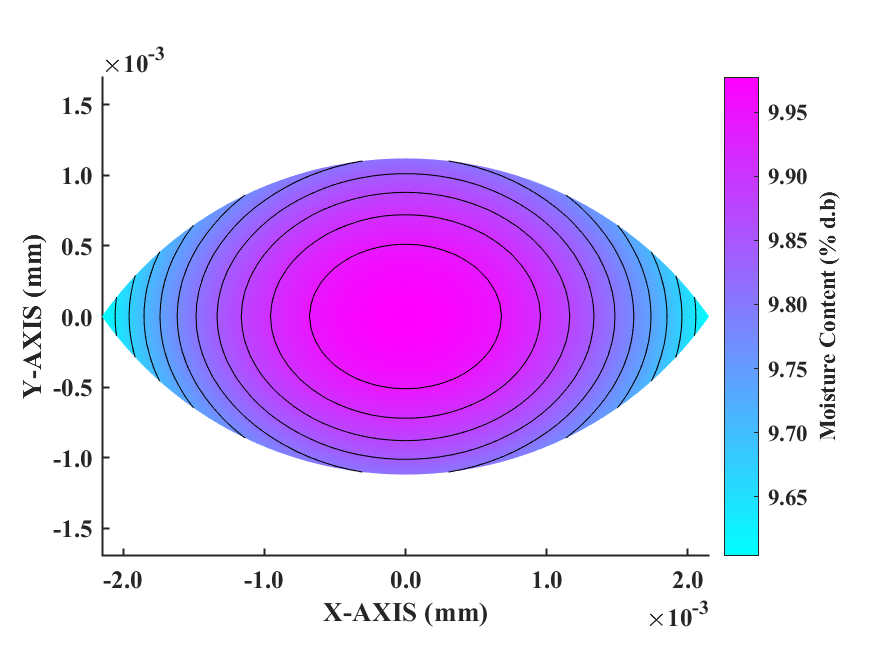

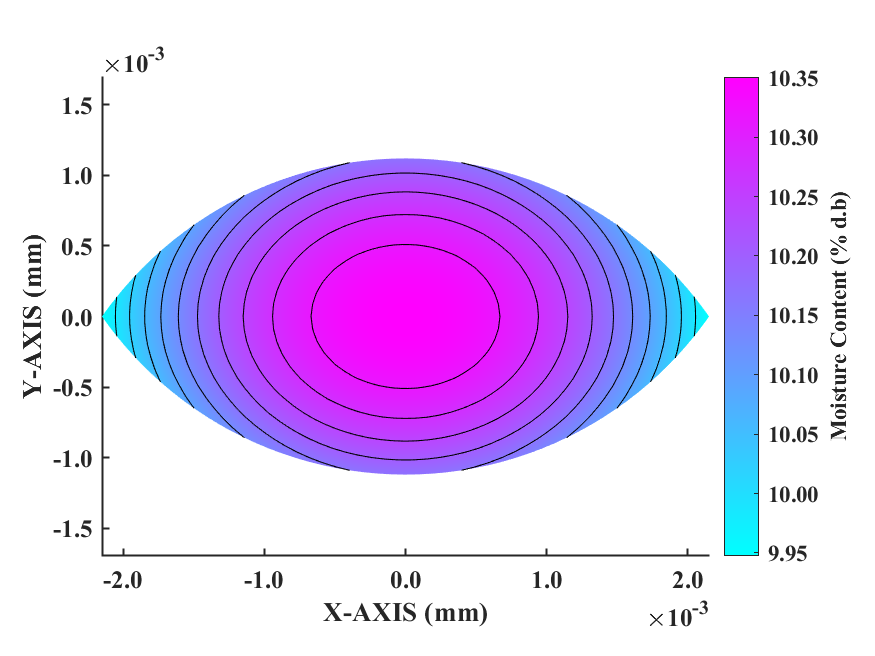

Supplement: Multimedia component 1 [file mmc1.docx]
